# The Role of Machine Learning in Knowledge-Based Response-Adapted Radiotherapy

**DOI:** 10.3389/fonc.2018.00266

**Published:** 2018-07-27

**Authors:** Huan-Hsin Tseng, Yi Luo, Randall K. Ten Haken, Issam El Naqa

**Affiliations:** Department of Radiation Oncology, University of Michigan, Ann Arbor, MI, United States

**Keywords:** adaptive radiotherapy, personalized treatment, deep learning, statistical learning, big data

## Abstract

With the continuous increase in radiotherapy patient-specific data from multimodality imaging and biotechnology molecular sources, knowledge-based response-adapted radiotherapy (KBR-ART) is emerging as a vital area for radiation oncology personalized treatment. In KBR-ART, planned dose distributions can be modified based on observed cues in patients’ clinical, geometric, and physiological parameters. In this paper, we present current developments in the field of adaptive radiotherapy (ART), the progression toward KBR-ART, and examine several applications of static and dynamic machine learning approaches for realizing the KBR-ART framework potentials in maximizing tumor control and minimizing side effects with respect to individual radiotherapy patients. Specifically, three questions required for the realization of KBR-ART are addressed: (1) what knowledge is needed; (2) how to estimate RT outcomes accurately; and (3) how to adapt optimally. Different machine learning algorithms for KBR-ART application shall be discussed and contrasted. Representative examples of different KBR-ART stages are also visited.

## 1 Introduction

Recent advances in cancer multimodality imaging (CT/PET/MRI/US) and biotechnology (genomics, transcriptomics, proteomics, etc.) have resulted in tremendous growth in patient-specific information in radiation oncology, ushering in the new era of Big Data in radiotherapy. With the availability of the individual-specific data, such as clinical, dosimetric, imaging, molecular markers, before and/or during radiotherapy (RT) courses, new opportunities are becoming available for personalized radiotherapy treatment (1, 2).

The synthesis of this information into actionable knowledge to improve patient outcomes is currently a major goal of modern radiotherapy (RT). Subsequently, knowledge-based response-adapted radiotherapy (KBR-ART) has emerged as an important framework that aims to develop personalized treatments by adjusting dose distributions according to clinical, geometrical changes, and physiological parameters observed during a radiotherapy treatment course. The notion of KBR-ART extends the traditional concept of adapted RT (ART) (3, 4), primarily based on imaging information for guidance, into a more general ART framework that can receive and process all relevant patient-specific signals that can be useful for adaptive decision-making. Our goal is to explore in more details the processes involved in the KBR-ART framework that would allow aggregating and analyzing relevant patient information in a systematic manner to achieve more accurate decision making and optimize long-term outcomes.

**Figure 1:**
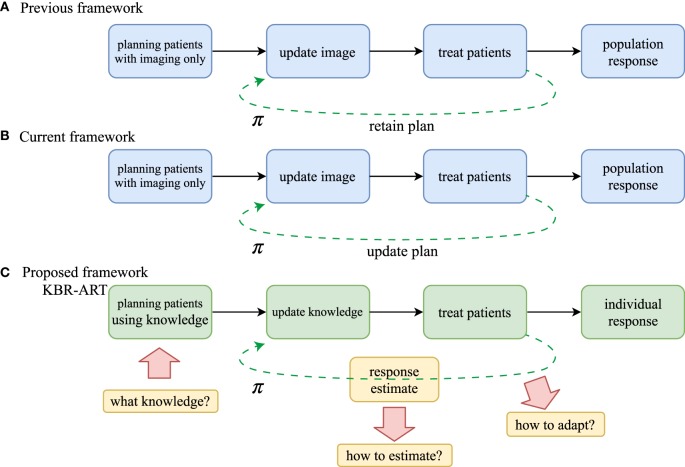
Comparison of workflow of **(A)** non-adaptive RT, **(B)** current image-based ART, and **(C)** the proposed KBR-ART approach. The current ART **(B)** mostly relies on image guidance such as computed tomography (CT), positron emission tomography (PET), and magnetic resonance imaging (MRI). In KBR-ART, the planning patients stage can utilize general knowledge about patient status (imaging + biological markers) as information for adapting treatment instead of using imaging only. Two major differences between previous/current RT and KBR-ART are that (1) knowledge is no longer restricted to imaging only and can include biological markers such as tumor genetics or blood-based inflammatory proteins (cytokines) to inform predictive modeling and decision-making; and (2) application process of machine learning for adapting a treatment plan π in KBR-ART.

The proposed KBR-ART framework can be thought of as being comprised of four stages, as depicted in Figure 1. These stages include: (1) *planning patients using available knowledge*, or pre-treatment modeling, (2) *updating the prediction models with evolving knowledge through the course of therapy*, or during-treatment modeling, (3) personalizing initial patient’s treatments, and (4) *adapting the initial treatment to individual’s responses*, where the two middle steps can be repeated at each radiation dose fraction (or few fractions) so that optimal treatment objectives are met and potentially long-term goals are optimized, i.e., long-term tumor control with limited side effects to surrounding normal tissues.

The first step in the implementation of a KBR-ART framework starts at the planning stage of patients by extending the current “image-only patients” into a more general preparation stage that can incorporate all relevant informatics signals for evaluating available treatment options, c.f. Figures 1A,B. Thus, the “K” in our KBR-ART refers to any useful knowledge (e.g., imaging (CT/PET/MRI) and biological markers (genomics, transcriptomics, proteomics, etc.) that can potentially aid the process of personalizing treatment to an individual patient’s molecular characteristics and is not limited to imaging only as currently is the case. In Section 2, we shall introduce four major categories of data that are relevant to improved knowledge synthesis in RT. As the era of Big data (BD) is upon us, many useful tools applied for BD analytics are being actively developed in the context of modern machine learning algorithms, where KBR-ART is expected to be a prime beneficiary of this progress toward the development of dynamically personalized radiotherapy treatment leading to better outcomes and improved patients’ quality of life. However, there are three essential questions pertaining to the successful development of a KBR-ART framework in radiotherapy that need to be addressed:

Q1: What knowledge should be synthesized for radiotherapy planning?
Q2: How can we develop powerful predictive outcome modeling techniques based on such knowledge?
Q3: How can we use these models in a strategically optimal manner to adapt a patient’s treatment plan?

**Figure 2:**
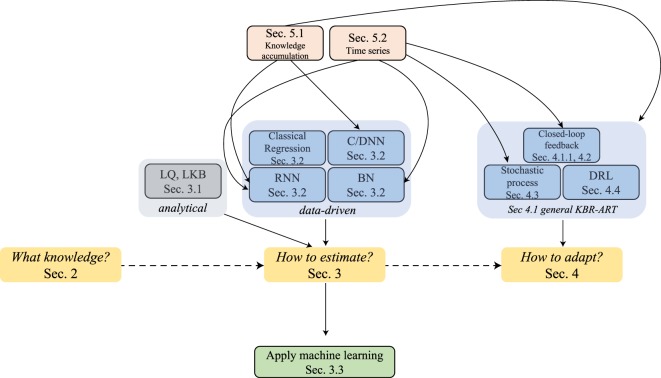
The graph outlines the sectional relationships and the organization of this paper, where the main thread is through Sections 2–4 can address the three pertained questions involved in KBR-ART design and development, Figure 1C.

The answers to these three questions are at the core of successful development of the proposed KBR-ART framework and we shall attempt to address them in more depth in Sections 2–4 of this paper. During the process of exploring the answers to these questions, we shed more light on the pivotal role that machine learning algorithms play in the design and development of a modern KBR-ART system in subsequent sections as outlined in Figure 2.

A major inherent merit of the KBR-ART framework is that the treatment planning would be designed to dynamically adapt to ongoing changes during the course of therapy to optimize radiotherapy goals of eradicating the tumor while minimizing harm to uninvolved normal tissue based on the individual patient’s characteristics. As shown in Figure 1, adaptation of a treatment plan can be more formally accomplished in accordance to a decision making function *π*. This is represented in Figure 1A for the previous/current framework, where *π* is a non-varying function but in the case of KBR-ART, Figure 1B, *π* is a time-varying function that depends on the information (knowledge updates) available during the course of therapy. The following scenario may be used as an example on how KBR-ART can be implemented in practice: a given planned radiation course was considered optimal according to an initial population-based model such as traditional dose-based tumor control probability (TCP) and normal tissue complication probability (NTCP) and the goal is to optimize the uncomplicated tumor control [*p*^+^ = TCP ⋅ (1–NTCP)], for instance. Then, through the course of fractionated radiotherapy treatment, the patient did not achieve the predicted TCP value as expected, or worse suffered from unexpected toxicities due to treatment, i.e., NTCP exceeded the designed risk limit. This is where KBR-ART comes into action; to learn from current observations with its previous decisions taking into account available information during therapy and to adjust the course of action [e.g., increase dose to improve TCP or decrease it to specific organ-at-risk (OAR) to limit its NTCP] and develop a better personalized treatment plan based on the updated knowledge (from imaging and biomarkers) of the specific patient under treatment as shown in Figure 1B.

Much effort of this study will be devoted to tackling questions (ii and iii), which requires consideration of some advanced data-driven models that can also incorporate temporal information (i.e., knowledge updates). The steps involved in the development of a knowledge-adapted plan using the KBR-ART framework will be the main subject of this paper. For this purpose, we will first review pertained modern machine learning algorithms that feature modeling of sequential data. These include efficient *deep-learning* approaches such as convolutional neural networks (CNNs), recurrent neural networks (RNNs), and the more recently developed deep reinforcement learning (DRL). The subject of sequential data modeling have been applied in many diverse fields, such as handwriting recognition (5), speech recognition (6), bioinformatics (7), medical care (8, 9), and also high energy physics (10).

The introduced algorithms based on deep learning would require some basic background of neural networks (NNs) which are briefly reviewed in Section 3.2.2. Most of the notations in this paper are self-contained and self-consistent. In addition to the presented advanced data-driven models, we also provide probabilistic and statistical perspectives as a theoretical foundation for sequential machine learning models. In particular, via “*filtration*” we are to describe notions related to “knowledge accumulation” or “growing of knowledge” in more concrete manner. A main part of KBR-ART development relies on constructing a new RT plan prescription based on historical information; thus we would like to address issues related to representing knowledge accumulation in sequential learning models.

**Figure 3:**
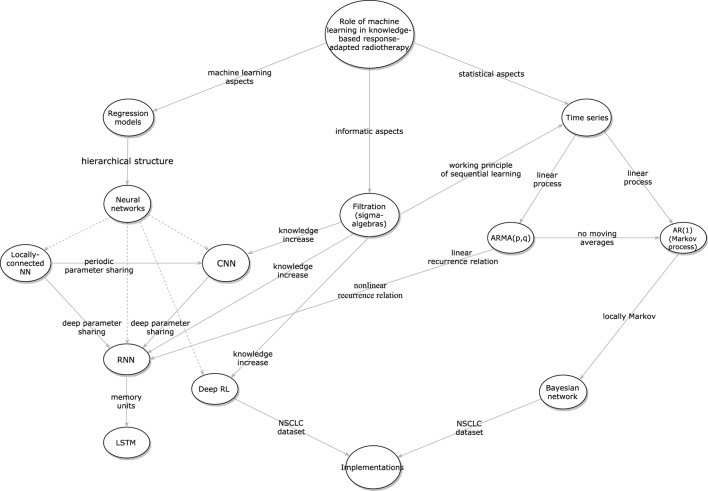
The inter-relations between the different presented algorithms for designing KBR-ART a framework.

Moreover, we recognize that KBR-ART has a close analogy to stock pricing or autonomous car driving, in that it shares the same goal of analyzing acquired information a long a period of time to maximize final rewards (e.g., better radiotherapy treatment outcome in our case). Therefore, techniques derived from time series analysis will be helpful to analyze such sequential data from an analytical perspective, such as the trends and the stationarity of such stochastic (random) processes. In particular, it suffices for our purpose to revisit main *linear processes*, such as the autoregressive moving average (ARMA) model and its natural descendant the autoregressive (AR) models, which can be linked to Bayesian networks (BNs), another useful approach for dynamical learning as summarized in Figure 3. Together, our goal is to provide a comprehensive overview and a frontier survey that covers the major facets for the application of KBR-ART and layout the foundation for this emerging field.

It worth noticing that we organized the sections of this paper so that it follows the necessary building steps for the development of a successful KBR-ART framework as pertained to addressing the three aforementioned questions involved in KBR-ART implementation and review the related literature accordingly. Two implementations using non-small lung cancer (NSCLC) datasets will be presented for illustration.

## 2 Q1: What Knowledge to be Used for KBR-ART Planning?

There are four major types of RT data that are potentially useful as part of the knowledge synthesis for KBR-ART: *clinical, dosimetric, imaging radiomics, and biological data*. To understand why and how they can be informative for assessing treatment outcomes, we provide a brief description about these four categories of data.

### 2.1 Clinical Data

Clinical data refers to cancer diagnostic characteristics (e.g., grade, stage, histology, site, etc.), physiological metrics (e.g., blood cell counts, heart/pulse rates, pulmonary measurements, etc.), and patient-related information (e.g., comorbidities, gender, age, etc.). Due to their nature, clinical data can usually be found in unstructured format such that can be challenging for extracting information directly. Therefore, machine learning techniques for natural language processing could be useful for transforming such data into structured format (e.g., tabulated) before further processing (11).

### 2.2 Dosimetric Data

Dosimetric data are informatic to the treatment planning process in RT, which includes simulated calculation of radiation dose using computed tomography (CT) imaging. In particular, dose–volume metrics obtained out of dose–volume histograms (DVHs) are extensively investigated for outcome modeling (12–16). Useful metrics are typically the volume receiving greater than or equal to a certain dose (*Vx*), the minimum dose to the hottest *x*% of the volume (*Dx*), mean, maximum, minimum dose, etc. (17). Notably, a dedicated software based on MATLAB™ called “DREES” can derive theses metrics automatically and apply them in outcome prediction models of RT response (18).

### 2.3 Radiomics Data

Radiomics is a field of medical imaging study that aims to extract meaningful quantitative features from medical images and relate this information to clinical and biological endpoints. The most common imaging modality is CT, which has been considered the standard for treatment planning in RT. Other imaging modalities used for improving treatment monitoring and prognosis in various cancer types are also used, such as positron emission tomography (PET), and magnetic imaging resonance (MRI). These modalities can be used individually or combined (19, 20).

### 2.4 Biological Data

According to (21) a biomarker is defined as *“a characteristic that is objectively measured and evaluated as an indicator of normal biological processes, pathological processes, or pharmacological responses to a therapeutic intervention*.*”* Measurements of biomarkers are typically based on tissue or fluid specimens, which are analyzed using molecular biology laboratory techniques (22) and have the following two categories according to their biochemical sources:

(a) *Exogenous biomarkers*: by injecting foreign substance into patients such as that used in molecular imaging and are used in radiomics applications.
(b) *Endogenous biomarkers*: there exists two subclasses within this category:
  (i) *Expression biomarkers*: changes measured in protein levels or gene expression.
  (ii) *Genetic biomarkers*: measuring variations between the underlying DNA genetic code and tumors or normal tissues.

### 2.5 Example: Aggregating Relevant Knowledge From a Lung Cancer Dataset

In this paper, we shall apply an institutional non-small cell lung cancer (NSCLC) dataset (23) as an example for implementation of KBR-ART. The first step is to collect relevant knowledge from such dataset that is suitable for the purposes of adapting radiotherapy treatment planning during a fractionated course. These data will be used subsequently for outcome modeling (TCP/NTCP) and plan adaptation as discussed later.

#### 2.5.1 Data Description

The NSCLC dataset was recorded from NSCLC patients, where they have been treated on prospective protocols with standard and dose escalated fractionation under IRB approval (24). Collectively, 125 patients with relatively complete characteristics were selected for predicting TCP (local control) and NTCP (radiation pneumonitis of grade 2 or above (RP2)).

The dataset had over 250 features containing positron emission tomography (PET) imaging radiomics features, circulating inflammatory cytokines, single-nucleotide polymorphisms (SNPs), circulating microRNAs, clinical factors, and dosimetric variables before and during radiotherapy. All features were recorded at three time periods (at baseline, at 2 weeks of treatment, and at 4 weeks). However, certain features were collected only at baseline such as microRNAs and SNPs. Thus, the data for the purpose of KBR-ART can be represented as forming 3 time blocks:

(1) $$\begin{array}{l} N\text{ samples}\\ \{\begin{array}{ccc} {}[\begin{array}{cccc} x_{11}^{\left(0\right)} & x_{12}^{\left(0\right)} & \ldots & x_{1n}^{\left(0\right)}\\ x_{21}^{\left(0\right)} & x_{22}^{\left(0\right)} & \ldots & x_{2n}^{\left(0\right)}\\ \vdots & \vdots & \vdots & \vdots \\ x_{N1}^{\left(0\right)} & x_{N2}^{\left(0\right)} & \ldots & x_{Nn}^{\left(0\right)} \end{array} & \left|\begin{array}{cccc} x_{11}^{\left(1\right)} & x_{12}^{\left(1\right)} & \ldots & x_{1n}^{\left(1\right)}\\ x_{21}^{\left(1\right)} & x_{22}^{\left(1\right)} & \ldots & x_{2n}^{\left(1\right)}\\ \vdots & \vdots & \vdots & \vdots \\ x_{N1}^{\left(1\right)} & x_{N2}^{\left(1\right)} & \ldots & x_{Nn}^{\left(1\right)} \end{array}\right| & \begin{array}{cccc} x_{11}^{\left(2\right)} & x_{12}^{\left(2\right)} & \ldots & x_{1n}^{\left(2\right)}\\ x_{21}^{\left(2\right)} & x_{22}^{\left(2\right)} & \ldots & x_{2n}^{\left(2\right)}\\ \vdots & \vdots & \vdots & \vdots \\ x_{N1}^{\left(2\right)} & x_{N2}^{\left(2\right)} & \ldots & x_{Nn}^{\left(2\right)} \end{array}] \end{array}, \end{array}$$

where $x_{\mathrm{ij}}^{\left(k\right)}$ denotes the value of the *j*th feature of patient *i* at time period *k*.

Values of mean tumor and lung doses were computed in their 2 Gy equivalents (EQD2) by using the linear-quadratic (LQ) model (Section 3.1.1) with *α*/*β* = 10 *Gy*, 4 *Gy* for the tumor and the lung, respectively. Generalized equivalent uniform doses (gEUDs) with various *a* parameters were also calculated for gross tumor volumes (GTVs) and uninvolved lungs (lung volumes exclusive of GTVs).

## 3 Q2: How to Estimate Radiotherapy Outcome Models from Aggregated Knowledge?

Radiotherapy outcome models are typically expressed in terms of tumor control probability (TCP) and normal tissue complication probability (NTCP) (25, 26). In principle, both TCP and NTCP may be evaluated using analytical and/or data-driven models. Though the former provides structural formulation, it can be incomplete and less accurate due to the complexity of radiobiological processes. On the other hand, data-driven models tend to learn empirically from the data observed, and thus they are capable of considering higher complexities and interactions of irradiation with the biological system. The trade-offs between analytical models and data-driven models can vary in terms of radiobiological understanding and prediction accuracy. In the following, we list examples, more detailed description on treatment outcome models can be found in (27).

### 3.1 Analytical Models

These models are generally based on simplified understanding of radiobiological processes and can provide a mechanistic formalism of radiation interactions with live tissue.

#### 3.1.1 TCP

The most prevalent TCP models are based on the linear quadratic (LQ) model (28) parametrized by the radiosensitivity ratio *α/β* derived from clonogenic cell survival curves. The LQ model expresses the survival fraction (SF) after irradiation as follows:

(2) $$\text{SF}=e^{-\alpha D-\beta D^{2}},$$

where *D* ≥ 0 is the total delivered dose. For *n* fractions of dose *d* in uniformly delivered fractions is represented by:

(3) $$\text{SF}=e^{-n\left(\alpha d+\beta d^{2}\right)}.$$

Many types of TCP models were proposed (28) in the literature such as the birth-death (29) and the Poisson-based (30) models, which are expressed as:

(4) $$\text{TCP}=e^{-N\cdot e^{-n\left(\alpha d+\beta d^{2}\right)-t\text{ ln }2∕T_{\text{pot}}}},$$

where *N* is the initial number of colonogenic cells, and *T*_pot_ denotes the potential cell doubling time, with *t* as the time difference within the total treatment elapse *T*, the lag period before accelerated clonogenic repopulation begins.

#### 3.1.2 NTCP

**Figure 4:**
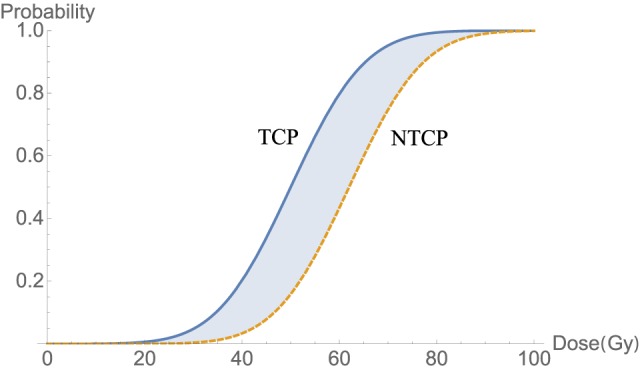
An illustration of a therapeutic ratio showing that the trade-off between TCP and NTCP as delivered dose increases. The blue-shaded area between two curves TCP (blue) and NTCP (orange-dashed) is a best window for dose delivery.

The most frequently used analytical model is the Lyman–Kutcher–Burman (LKB) model, which is a phenomenological approach (31). In the uniform dose case, NTCP is expressed by a gaussian integral (probit function):

(5) $${\text{NTCP}}_{m,D_{50}}\left(x\right)=\frac{1}{\sqrt{2\pi }}\;\int_{x}^{-\infty }\;e^{-u^{2}/2}\;\mathrm{du},\;\left(x=\frac{D-D_{50}}{m\;D_{50}}\right),$$

where *D*_50_ is defined as the dose that corresponds to NTCP probability (curve in Figure 4) of 50% and *m* is a parameter tuning the shape of the NTCP curve. Typical trade-off between TCP and NTCP to achieve a therapeutic ratio is shown in Figure 4.

To account for dose inhomogeneities in developing TCP/NTCP models, the Equivalent Uniform Dose (EUD) (32) or Generalized EUD (gEUD) (33) are used. Mimicking a weighted sum of doses, gEUD is given by:

(6) $$\text{gEUD}=\sqrt[{a}]{\underset{i=1}{\overset{n}{\sum }}\;v_{i}\;D_{i}^{a}},$$

where *v_i_* is the fractional organ volume receiving dose *D_i_* and *a* is a volume parameter that depends on the tissue type. An *a* < 0 value will correspond to minimum dose effect, which is typically associated with tumor response. An *a* > 0 value will correspond to maximum dose effect, which is typically associated with serial normal tissue architecture response, while an *a* = 1 will correspond to mean dose effect, which is associated with parallel normal tissue architecture response.

More complex analytical models for toxicity can be developed by incorporating variables other than dose in the LKB model, for instance (34, 35):

(7) $${\text{NTCP}}_{m,D_{50},\text{DMFs}}\left(x\right)=\frac{1}{\sqrt{2\pi }}\;\int_{x}^{-\infty }\;e^{-u^{2}∕2}\;\mathrm{du}$$

with

$$x=\frac{D_{\text{eff}}\cdot {\text{DMF}}_{1}\cdot {\text{DMF}}_{2}\cdots {\text{DMF}}_{k}-D_{50}}{m\;D_{50}},$$

where the DMFs are dose modifying factors and represent the impact of covariates other than dose (e.g., single-nucleotide polymorphism (SNPs) genotype, copy number variations (CNVs), smoking status, etc.). Although analytical models are useful, in many circumstances, they are simply approximations of the complex physical and biological processes that are currently beyond such simple formalisms. Therefore, more data-driven approaches are being sought to achieve more accurate predictions of TCP/NTCP.

### 3.2 Data-Driven Models

By definition, data-driven models are approximations built based on observation of data. However, one drawback is that such modeling is likely not unique even from the same dataset and, therefore, one needs to choose a suitable technique that fits one’s dataset best, which is an open question in the data science world. The purpose main of this section is to present several advanced data-driven techniques that can suite the implementation of predictive outcome modeling component of the KBR-ART framework. Below, we summarize some frequently used data-driven techniques for outcome modeling ranging from classical regression models to more advanced machine learning techniques.

#### 3.2.1 Classical Models

Regression models such as Ridge, LASSO, and Logistic are commonly used foe building outcome models and follow conventional statistical approaches (36). They are essentially constructed by minimizing the following objective:

(8) $$L\left(\text{w}\right)=\sum_{i=1}^{N}\;{\left[{\text{y}}_{i}-\left(\left\langle \text{w},{\text{x}}_{i}\right\rangle +b\right)\right]}^{2}+\text{λ}\;\cdot \;h\left(\text{w}\right),$$

where ${\text{x}}_{i}\in R^{n}$ and ${\text{y}}_{i}\in R$, *i* = 1, … , *N*, are the data input and outputs, respectively. Here, the weights $\text{w}\in R^{n}$ and bias b∈R are unknown parameters to be fitted by minimizing **regression error**, Equation (8). The second term in Equation (8) represents **penalty**, usually used to suppress possible model’s overfitting. There are several types of penalty corresponding to different model characteristics, such as *h*(**w**) = ∥**w**∥ is called the **LASSO** by Tibshirani (37), *h*(**w**) = ∥**w**∥^2^ is called the **Rigid (Tikhonov) regularization** (37), and $h\left(\text{w}\right)\;=\;{\text{λ}}_{1}∥\text{w}∥\;+\;{\text{λ}}_{2}{∥\text{w}∥}^{2}$ is called the **Elastic Net regularization (38)**. The regularization parameter λ controls the magnitude of the penalty.

Due to the characteristic of *L*_1_-norm, ||⋅||, the LASSO regularization tends to suppress many parameters to equal zero, so that the parameter vector is sparse, which makes it a natural candidate for relevant *feature selection* (39).

Another benefit of regression models other than their simplicity is the convex optimization property of their loss function, which guarantees optimal fitting parameters **w** = **w**_∗_. In fact, it can be explicitly solved using simple matrix inversion **w**_∗_ = (**X**^*T*^**X** + λ*I*)^−1^⋅**X***^T^* **y**, for Ridge regression, for instance, where **X** is known from the given data:

(9) $$\text{X}\overset{\mathrm{def}}{=}\left(\begin{array}{ccc} - & {\text{x}}_{1} & -\\ - & {\text{x}}_{2} & -\\ & \vdots & \\ - & {\text{x}}_{N} & - \end{array}\right)=\left(\begin{array}{cccc} 1 & x_{11} & \cdots & x_{1n}\\ 1 & x_{21} & \cdots & x_{2n}\\ \vdots & \vdots & \ddots & \vdots \\ 1 & x_{N1} & \cdots & x_{\mathrm{Nn}} \end{array}\right).$$

#### 3.2.2 Neural Networks

**Figure 5:**
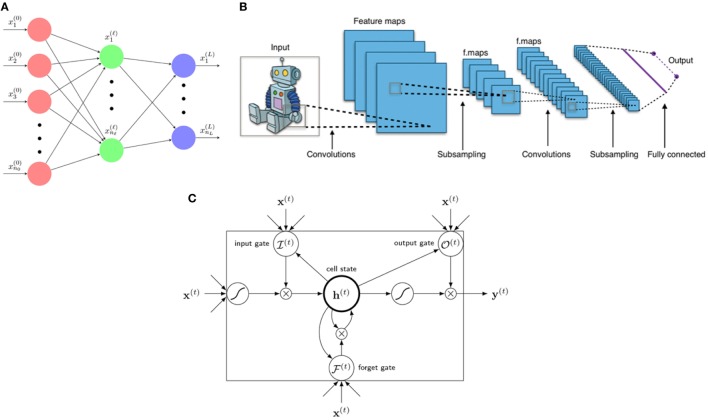
Three main architectures of deep learning. **(A)** A neural network is a composition function interpreted to have several layers from 0, … , *L* with neural nodes (*n*_0_, … , *n*_ℓ_, … , *n_L_*). Variables **x**^(ℓ)^ = $\left({\text{x}}_{\text{1}}^{\left(\ell \right)},\ldots ,{\text{x}}_{{\text{n}}_{\ell }}^{\left(\ell \right)}\right)$ are called neurons of layer ℓ. **(B)** A typical architecture of a CNN consists of several layers including linear convolutional layers, pooling layers, and a final fully connected layer for classification (or regression). A kernel (filter) acts as a mask operating only on neighboring information (pixels) yet blocking distant information. [Figure created by Aphex34 distributed under a CC BY-SA 4.0 license (from Wikimedia Commons)]. **(C)** An LSTM unit consists of 1 cellstate **h**^(*t*)^ and 3 gates: forget gate $F^{\left(t\right)}$, input gate $I^{\left(t\right)}$, and output gate $O^{\left(t\right)}$, with **x**^(*t*)^ as input and **y**^(*t*)^ as final (prediction) output.

One notable model in machine learning is called **Neural Networks** (NN), which are inspired by the neurobiology of the brain, and hence the name. Mathematically, NNs utilize (repeated) composition of nonlinear transformations in developing their architecture. The definition is fairly simple (40); given a set of data inputs ${\text{x}}_{i}\in R^{n}$ and labels ${\text{y}}_{i}\in R$, *i* = 1, … , *N* as defined above, a NN is aimed to approximate a function of the form:

(10) $$f_{\text{w},\text{b}}\left(\text{x}\right)=\sigma_{L}\left({\text{w}}^{\left(L\right)}\cdot \sigma_{\left(L-1\right)}\left({\text{w}}^{\left(L-1\right)}\cdot \cdots \sigma_{1}\left({\text{w}}^{\left(0\right)}\cdot \text{x}+{\text{b}}^{\left(0\right)}\right)+\;{\text{b}}^{\left(L-2\right)}\right)+{\text{b}}^{\left(L\right)}\right),$$

via adjusting unknown coefficients ${\left\{{\text{w}}^{\left(\ell \right)}\in R^{n_{\ell }\times n_{\ell +1}}\right\}}_{\ell =0}^{L}$ and ${\left\{{\text{b}}^{\left(\ell \right)}\in R^{n_{\ell }}\right\}}_{\ell =0}^{L}$ such that the loss function is minimal between the data and the NN model:

(11) $$L\left(\left\{{\text{w}}^{\left(\ell \right)}\right\},\left\{{\text{b}}^{\left(\ell \right)}\right\}\right)=\underset{i=1}{\overset{N}{\sum }}\;g\left({\text{y}}_{i},f_{\text{w},\text{b}}\left({\text{x}}_{i}\right)\right),$$

where in Equation (10), the given functions $\sigma_{\ell }:\;R^{n_{\ell }}\to R^{n_{\ell }}$ are called *activation functions*, which are fixed for a particular architecture. The integer *L* of max composition is interpreted as layers with index ℓ = 0, … , *L* denoting the layer number as shown in Figure 5A and *n_ℓ_* is an integer denotes the number of **nodes (neurons)** in layer *ℓ*. The function *g* in Equation (11) should also be fixed depending on data query type. For continuous labels **y***_i_*, such NN is called a regression prediction function with $g\left(\text{y},\text{h}\left(\text{x}\right)\right)\;=\;||\text{y}\;-\;h\left(\text{x}\right)|{|}^{2}$ typically adopted for an arbitrary loss function $\text{h}:R^{n}\to R^{m}$. For discretized labels of multidimensions **y** = (*y*_1_, … , *y_m_*), such NN is called a classification prediction function with cross entropy loss function $g\left(\text{y},\text{h}\left(\text{x}\right)\right)=\sum_{k=1}^{m}\;\left[y_{k}\log\left(h_{k}\left(\text{x}\right)\right)+\left(1-y_{k}\right)\log\left(1-h_{k}\left(\text{x}\right)\right)\right]$ typically chosen with **h** = (*h*_1_, … , *h_m_*).

In practice, there are several choices for activation functions *σ*_i_, such as sigmoid, ReLu, eLu, Leaky ReLU function, etc., whose effectiveness usually depends on the nature of the dataset and the problem in question. The terms relating *forward dynamics, error backward propagation*, and *weights gradient descent* are technical procedures for estimating the unknown coefficients ${\left\{{\text{w}}^{\left(\ell \right)}\in R^{n_{\ell }\times n_{\ell +1}}\right\}}_{\ell =0}^{L}$ and ${\left\{{\text{b}}^{\left(\ell \right)}\in R^{n_{\ell }}\right\}}_{\ell =0}^{L}$ from Equation (11). Although the design construction of an NN is relatively simple, the proper optimization of its parameters could be tedious numerically (40, 41).

In general, it is conventionally dubbed a *deep neural network* (DNN) when the number of hidden layers exceed 2, or *L* ≥ 3. These neural networks are widely applied and are the foundations for the emerging field of *deep learning*, which is currently overperforming many of the classical machine learning techniques.

#### 3.2.3 Deep learning Models

In KBR-ART, one expects that the processes involved in outcome modeling and adaptation procedures can be quite complex in nature for individualizing patient’s treatment according to her/his predicted response over the course of fractionated therapy. There are few advanced data-driven models, mostly deep learning based, which can effectively into consideration such temporal information for updating knowledge and interactions between physical and biological variables for adapting therapy. In the following, we will briefly describe some of the main deep learning technologies in the literature.

##### 3.2.3.1 Convolutional Neural Networks (CNNs)

CNNs are best known for image recognition and image-related prediction. The idea of CNN stemmed from the successful application of the signal processing operation of *convolution* in imaging processing, which was then been applied into neural networks for handling image related tasks. A CNN typically consists of several *convolutional layers, pooling layers, with activation functions* (42), where the convolution layer is the core component that applies an efficient convolutional filter (kernel) to the data in contrast to the tedious matrix operations described earlier with standard NN. In the case of a 2D image of size *L*_1_ × *L*_2_ with multi color channels (*C*_1_), the data are represented by a 3d-tensor $I={\left\{I_{i,j,\alpha }\right\}}_{i=1,j=1,\alpha =1}^{L_{1},L_{2},C_{1}}\in R^{2+1}$, a convolutional layer with stride *s* renders an output image (also called **feature maps**) $\tilde{I}$ (of size ${\tilde{L}}_{1}\times {\tilde{L}}_{2}$ with *C*_2_ channels) by applying the following convolution process (42).

(12) $$\begin{array}{c} {\tilde{I}}_{k,\ell ,\beta }=\sum_{m,n,\alpha }^{L_{1},L_{2},C_{1}}\;w_{m,n,\alpha ,\beta }\cdot I_{s\left(k-1\right)+m,s\left(\ell -1\right)+n,\alpha }\\ \left(\text{image convolution}.k=1,\ldots ,{\tilde{L}}_{1},\ell =1,\ldots ,{\tilde{L}}_{2},\beta =1,\ldots C_{2}\right) \end{array},$$

here, $\text{w}={\left\{w_{m,n,\alpha ,\beta }\right\}}_{m=1,n=1,\alpha =1,\beta =1}^{L_{1},L_{2},C_{1},C_{2}}\;\in \;R^{2+1+1}$ is a 4-tensor convolutional kernel. Such convolution process with stride is then equivalent to a regular convolution with image downsampling procedure. In fact, one can recognize that CNNs use these kernels in a neural network to “capture” local information within a neighborhood while “blocking” distant information or less related ones, as depicted in Figure 5B. Activation functions in CNNs have similar choices as a standard NN, Equation (10), mentioned above. CNNs has been successfully applied for image segmentation (43–46) in radiotherapy and for modeling of rectal toxicity in cervical cancer using transfer learning (47, 48). This will be further discussed in Section 3.3.1.

##### 3.2.3.2 Recurrent Neural Networks (RNNs)

RNNs are another variant of neural networks especially useful for learning sequential data, such as voice, text data, and handwriting. Therefore, it is also considered ideal for sequential adaptive radiotherapy with changing dose fractionations. In this case, suppose that we have sequential data $\left\{{\text{x}}^{\left(t\right)}\in R^{n}|\;t\in T\right\}$ as an input and $\left\{{\tilde{\text{y}}}^{\left(t\right)}\in R^{m}|\;t\in T\right\}$ as the corresponding labels where *T* denotes an index set (continuous or discrete) labeling separation across time steps. An important property of a RNN is that it introduces hidden units $\left\{{\text{h}}^{\left(t\right)}\in R^{k}|\;t\in T\right\}$ for making neural network deeper in increasing sequential prediction. A RNN is then aimed to learn the relationships between data $\left\{{\text{x}}^{\left(t\right)}\in R^{n}\right\}$ and labels $\left\{{\tilde{\text{y}}}^{\left(t\right)}\right\}$ via hidden units $\left\{{\text{h}}^{\left(t\right)}\in R^{k}\right\}$ dynamically.

An RNN is designed to model the hidden variables via the recursive function $f_{\theta }:R^{k}\times R^{n}\to R^{k}$.

(13) $${\text{h}}^{\left(t\right)}=f_{\theta }\left({\text{h}}^{\left(t-1\right)},{\text{x}}^{\left(t\right)}\right)\in R^{k},$$

where *θ* usually serves as unknown neural weights to be solved, as ${\left\{{\text{w}}^{\left(\ell \right)},{\text{b}}^{\left(\ell \right)}\right\}}_{\ell =0}^{L}$ in Equation (10).

One of the most successful RNN is the Long Short-Term Memory (LSTM). An LSTM is a state-of-the-art RNN model effective in sequential learning utilizing the so-called *gated units*, who learns by itself to store and forget internal memories when needed such that it is capable of creating long-term dependencies and paths through time, Figure 5C. A LSTM is constructed by 3 gates and 1 cell (hidden) state built up by the following equations.

(14) $$\begin{array}{l} F^{\left(t\right)}=\sigma_{g}+\left(W_{F}\cdot {\text{x}}^{\left(t\right)}+U_{F}\cdot {\text{h}}^{\text{(}t-\text{1)}}+{\text{b}}_{F}\right)\in \left[0,1\right]\\ I^{\left(t\right)}=\sigma_{g}+\left(W_{I}\cdot {\text{x}}^{\left(t\right)}+U_{I}\cdot {\text{h}}^{\text{(}t-\text{1)}}+{\text{b}}_{I}\right)\in \left[0,1\right]\\ O^{\left(t\right)}=\sigma_{g}+\left(W_{O}\cdot {\text{x}}^{\left(t\right)}+U_{O}\cdot {\text{h}}^{\text{(}t-\text{1)}}+{\text{b}}_{O}\right)\in \left[0,1\right]\\ {\text{h}}^{\left(t\right)}=F^{\left(t\right)}\circ {\text{h}}^{\left(t-1\right)}+I^{\left(t\right)}\circ \sigma_{\text{h}}\left(W_{\text{h}}\cdot {\text{x}}^{\left(t\right)}+U_{\text{h}}\cdot {\text{h}}^{\text{(}t-\text{1)}}+{\text{b}}_{\text{h}}\right)\\ {\text{y}}^{\left(t\right)}=O^{\left(t\right)}\circ \sigma_{\text{y}}\left({\text{h}}^{\text{(}t\text{)}}\right), \end{array}$$

where *σ_g_*, *σ*_**h**_, *σ*_**y**_ are 3 non-linear activation functions depending on one’s choice, $\left\{F^{\left(t\right)},I^{\left(t\right)},O^{\left(t\right)}\right\}$ are called the **forget gate**, **input gate**, and **output gate** at time *t*, respectively.

The 3 gates, with all their numerical values in [0,1], are used to control and determine when and how much should the previous information be kept or forgotten. The unknown parameters of an LSTM are (*W_h_, U_h_*, **b***_h_*) and $\left\{\left(W_{\alpha },U_{\alpha },{\text{b}}_{\alpha }\right)|\;\alpha =F,I,O\right\}$ and, therefore, an LSTM unit generally possesses four times parameters than a plain neural net in Equation (10) requiring a large amount of data for training. RNNs have been evaluated in radiotherapy for respiratory motion management (49). An interesting approach combining RNN with CNN was used for pancreas segmentation on both CT and MRI datasets, which mitigated the problem of using spatial smoothness consistency constraints (50, 51).

The previously presented machine learning methods do not allow visualization of the system dynamics and act primarily as a black box mapping from the input to the output data and are referred to as *discriminant* models. Alternatively, system dynamics of mapping input to output data can be revealed using so-called *generative* models. A common example of such models is Bayesian networks, which will be discussed next.

#### 3.2.4 Bayesian Networks

Bayesian networks (BNs) are a class of probabilistic graphical models (GM) corresponding to directed acyclic graphs (DAGs), which are also named as belief networks. BNs combine graph theory, probability theory, computer science, and statistics to represent knowledge in an uncertain domain. They are popular in the societies of statistics, machine learning, and artificial intelligence. Especially, BNs are mathematically rigorous and intuitively understandable, which enable an effective way to represent and compute the joint probability distribution (JPD) over a set of random variables (52).

Each BN includes the sets of nodes and directed edges. While the former indicate random variables represented by circles, the latter display direct dependencies among these variables illustrated by arrows between nodes. In a BN, an arrow from node *X_i_* to node *X_j_* shows a statistical dependence between them, which indicates that a value of variable *X_j_* depends on that of variable *X_i_*, or variable *X_i_* “affects” *X_j_*. Also, their relationship can be described as follows: Node *X_i_* is a parent of *X_j_* and node *X_j_* is the child of *X_i_*. In general, the set of nodes that can be reached on a direct path from the node is named as the set of its descendants, and the set of nodes from which the node can be reached on a direct path is called as the set of its ancestor nodes (53).

The DAG structure guarantees that no node can be its own ancestor or its own descendant, which is of vital importance to the factorization of the JPD of a collection of nodes. A BN is designed to reflect a conditional independence statement, where each variable is independent of its nondescendants in the BN given its parents. This property is used to significantly reduce the number of parameters required to characterize the JPD of the variables. Especially, this reduction leads to an efficient way in computing the posterior probabilities given the evidence (52, 54, 55).

Moreover, the parameters of the BN are described in a manner following a Markovian property, where the conditional probability distribution (CPD) of each node only depends on its parents. These conditional probabilities are often represented by a table for discrete random variables to list the conditional probability that a child node takes on each of the feasible values from each combination of values of its parents. The joint distribution of a collection of variables can be obtained uniquely by these conditional probability tables (CPTs).

Generally, a BN *B* can be considered as a DAG that represents a joint probability density function over a set of random variables *V*. The BN is defined by a pair B = ⟨*G, ϕ*⟩, where *G* is the DAG whose nodes *X*_1_, *X*_2_, … , *X_n_* denotes random variables, and whose edges indicate the direct dependencies between them. The graph *G* includes independence assumptions, where each variable *X_i_* is independent of its nondescendants given its parents in *G*. The second component *ϕ* represents the set of parameters of the BN. This set contains the parameter *θ*(*x_i_*|*π_i_*) = *P_B_*(*x_i_*|*π_i_*) for each realization *x_i_* of *X_i_* conditioned on *π_i_*, which is the set of parents of *X_i_* in *G*. Then, *B* describes a unique JPD over *V*:

(15) $$P_{B}\left(X_{1},X_{2},\ldots ,X_{n}\right)=\prod_{i=1}^{n}P_{B}\left(X_{i}|\pi_{i}\right)=\prod_{i=1}^{n}\;\theta_{X_{i}|\pi_{i}},$$

where if *X_i_* does not have parents, its probability distribution is considered to be unconditional; otherwise it is conditional. Once the variable indicated by a node is observed, the node is considered as an evidence node; otherwise the node is treated as a hidden or latent node. Because of their generative nature, BNs have been widely applied for modeling radiotherapy errors (56, 57) and outcomes (58–62). This will be further discussed in Section 3.3.2.

### 3.3 Example Application of Machine Learning to Outcome Modeling

As examples of application of modern machine learning to outcome modeling, in the following, we discuss application of a discriminant modeling approach by CNN of rectal toxicity and a generative modeling approach by BN for lung toxicity.

#### 3.3.1 NTCP Modeling of Rectal Toxicity Using CNN

**Figure 6:**
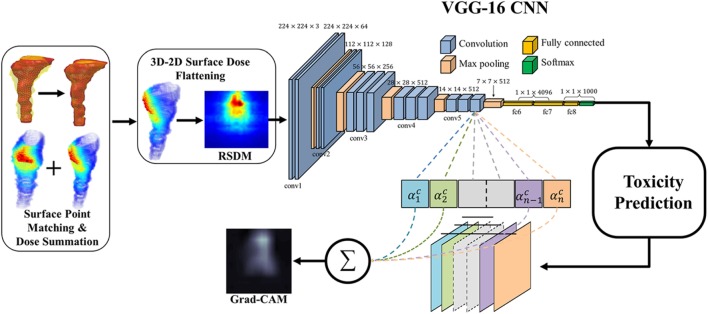
The workflow of the rectum toxicity study in (47) using VGG-16 receiving 2D RSDM image input with Grad-CAM map as interpretation of CNN weights. [© Institute of Physics and Engineering in Medicine. Reproduced by permission of IOP Publishing. All rights reserved.]

Zhen et al. (47) studied the possibility of modeling rectal toxicity in cervical cancer using CNNs from unfolded rectum surface dose maps (RSDMs) (63) with the help of transfer learning, as depicted in Figure 6. A retrospective data of 42 cervical cancer patients were studied. These patients were treated with external beam radiotherapy (EBRT) and/or brachytherapy (BT). The EBRT was delivered in 25 fractions (2 Gy/fraction) and BT was delivered in 4–6 fractions (6–7 Gy/frac).

**Figure 7:**
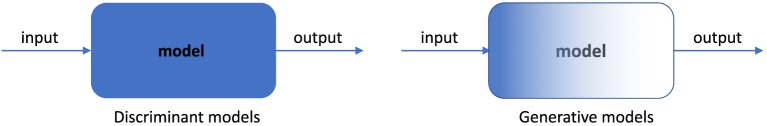
Comparison of discriminant and generative models, where the gradient coloring on generative models indicates the model transparency.

**Figure 8:**
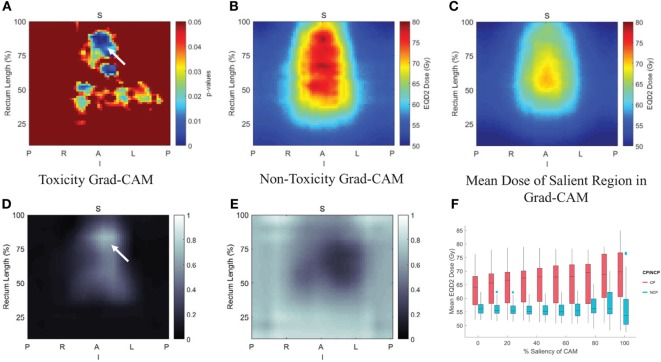
Pixelwise *p*-value map were shown in **(A)** with small *p* < 0.05, **(B,C)** are the average rectum RSDM of the toxicity and non-toxicity patients; and **(D,E)** are average Grad-CAM map of the toxicity and non-toxicity groups. **(F)** Box plot of the mean dose in different salient regions extracted from the Grad-CAM map. Details see (47).

For transfer learning, CNN of VGG-16 (64) was chosen as optimal architecture, which consists of 16 convolutional layers of suitable sizes including up to 138 million parameters. The VGG-16 is pretrained using a publicly annotated natural images database (ImageNet). The finetuned VGG-16 on the cervix cancer dataset with ADASYN method for imbalance correction, achieved an AUC of 0.89 on leave-one-out cross validation for rectal toxicity prediction. In addition to a successful model building of relating RSDMs to toxicity, Zhen et al. also attempted to interpret what and how CNNs “view” an RSDM, where the method of Grad-CAM map (65) was utilized to unveil the nature of the CNN learnt features (Figure 7). From Figure 8, one finds that the Grad-CAM interpreted maps (d, e) (from mapping CNN weights) have high consistency of distinct image patterns with toxicity (b) and non-toxicity (c) that were recognizable by human eyes. Therefore, by visualizing the CNN model, one can have better understanding of the features learned by the machine learning algorithm.

#### 3.3.2 NTCP Modeling of Lung Toxicity Using Bayesian Networks

Radiation pneumonitis of grade 2 or above (RP2) is a major radiation-induced toxicity in NSCLC radiotherapy, and it may depend on radiation dose, the patients’ clinical, biological, and genomic characteristics. In order to find appropriate treatment plans and improve patients’ therapeutic satisfaction, a systematic machine learning approach needs to be developed to find the most important features from the high dimensional dataset and to discover the relationships between them and RP2 for clinical decision-making. Thus, a BN approach was developed to explore interpretable biophysical signaling pathways influencing RP2 from a heterogeneous dataset including single nucleotide polymorphisms (SNPs), micro RNAs (miRNAs), cytokines, clinical data, and radiation treatment plans before and during the course of radiotherapy of NSCLC patients.

In this BN implementation, the dataset described in Section 2.5.1 with 79 patients (21 cases of RP2) was used for model building and 46 additional patients were reserved for independent model testing. The BN approach mainly included a large-scale Markov blanket (MB) method to select relevant predictors, and a structure learning algorithm to find the optimal BN structure based on Tabu search and the performance evaluation of outcome prediction (24). *K*-fold cross-validation was used to guard against over-fitting, and the area under the receiver-operating characteristics (AUC) curve was utilized as a prediction metric.

**Figure 9:**
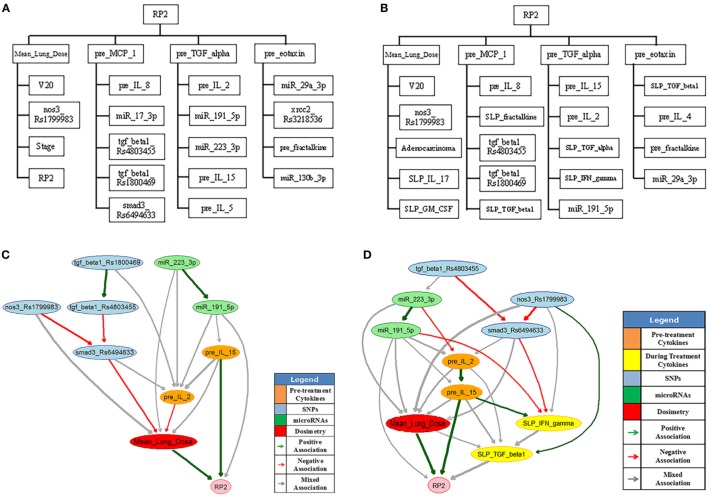
The extended MB neighborhoods of RP2 before **(A)** and during **(B)** radiation treatment, where the upper level shows the inner family of RP2 and the lower levels show the next-of-kin for each of its member. Pretreatment BN **(C)** and during-treatment BN **(D)** for RP2 prediction [figures reprinted with permission].

The large-scale MB method intends to identify the most relevant variables of RP2 before or during the course of radiotherapy. Figure 9A shows the extended MB neighborhoods of RP2 before radiation treatment, where the MB of RP2 based on pretreatment training data is formed from “Mean_Lung_Dose,” “pre_MCP_1,” “pre_TGF_alpha,” and “pre_eotaxin.” In the meantime, each of these variables has its own MB neighborhood as shown in Figure 9A. For example, “V20,” “nos3_Rs1799983,” “stage,” and “RP2” form the MB of “Mean_Lung_Dose.” In this study, potential variables of the BN were identified from the extended MB neighborhoods within two layers of RP2. Figure 9B indicates the updated extended MB neighborhoods in an extended model after incorporating the slopes of cytokine levels before and during-treatment (SLP) as the patients’ responses during the radiation treatment. Although the MB of RP2 during the radiation treatment based on the whole training dataset keeps the same as that in Figure 9A, the MB of “Mean_Lung_Dose” has been updated, and it includes patients’ cytokine responses such as “SLP_IL_17,” “SLP_GM_CSF.” Figures 9C,D illustrate biophysical signaling pathways from the patients’ relevant variables to RP2 risk based on pretreatment and during BN model building, respectively. The results of internal cross-validation show that the performance of the BN yielded an AUC = 0.82, and it was improved by incorporating during treatment cytokine changes to AUC = 0.87. In the testing dataset, the pre- and during AUCs were 0.78 and 0.82, respectively. It turns out that the BN approach allows for unraveling of relevant biophysical features that lead to RP2 risk and prediction of RP2, and this prediction improved by incorporating during treatment information (24).

## 4 Q3: How to Adapt Plans in KBR-ART?

The precise estimation of treatment outcome is necessary step before deciding on the right course of action, since we desire to evaluate potential outcomes effects beforehand as we weigh the different alternatives for the best possible strategy (i.e., set of actions) to optimize the individual’s treatment response. This is in a simplistic sense no different than playing board games or chess when a player may evaluate a dozen of options before carrying out a move. Therefore, by assuming one can attain accurate prediction estimates of TCP and NTCP, as discussed in the previous section, then, the final question to address in the context of KBR-ART is how to optimally adapt the plan (e.g., increase the tumor fraction dose) to achieve improved outcomes.

A utility function is usually required to estimate the total effect of a treatment plan weighting on both positive outcomes and the possible side effects caused. In RT, an example utility function called *complication-free tumor control (P^+^)* can be used. The *P*^+^ measures the performance of a treatment at each stage based on combined TCP and NTCP under the form *P*^+^ = *U*(TCP, NTCP; *θ*) where *P*^+^ indicates *probability of a positive treatment outcome*. One linear form is particularly simple and effective (66) where:

(16) $$P^{+}=\text{TCP}\times \left(1-\text{NTCP}\right)$$

Notably, some other functional forms may be used as well, such as Equation (42).

In the practice of KBR-ART, if one has already synthesized relevant knowledge (clinical, dosimetric data, … etc.) from Section 2 with variables *x*_1_, … , *x_n_* as predictors and applied analytical/data-driven models in Section 3, then we can derive models of TCP and NTCP in the form TCP = *f*_TCP_ (*x*_1_, … , *x_n_*) and NTCP = *f*_NTCP_ (*x*_1_, … , *x_n_*) based on retrospective data such that the *P*^+^ response estimation function reads:

(17) $$P^{+}=U\left(f_{\text{TCP}}\left(x_{1},\ldots ,x_{n}\right),f_{\text{NTCP}}\left(x_{1},\ldots ,x_{n}\right);\theta \right)$$

With the response estimation defined by the *P*^+^ utility functions, next, we design a scheme for treatment adaptation. Machine learning based on reinforcement learning (RL) is a suitable approach for realizing plan adaptation as it can search over all possible decisions to maximize the *P*^+^ function as *rewards* and identify the best *policy* (e.g., dose per fraction) for the treatment planning.

### 4.1 Generalized KBR-ART Framework

The KBR-ART can be described by the following general formulation:

(18) $$\begin{array}{ll} & \left\{{\text{x}}^{\left(t\right)}\in R^{n}|t\in T\right\},\;\left\{{\text{y}}^{\left(t\right)}\in R^{m}|t\in T\right\},\;\left\{{\text{u}}^{\left(t\right)}\in R^{p}|t\in T\right\}\\ & \;L\left(\left\{{\text{x}}^{\left(t\right)}\right\},\left\{{\text{y}}^{\left(t\right)}\right\},\left\{{\text{u}}^{\left(t\right)}\right\};\theta \right),\;C\left(\left\{{\text{x}}^{\left(t\right)}\right\},\left\{{\text{y}}^{\left(t\right)}\right\},\left\{{\text{u}}^{\left(t\right)}\right\};\phi \right), \end{array}$$

where **x**^(*t*)^ is the state of a system at time *t* ∈ *T*, **y**^(*t*)^ is the observation of state **x**^(*t*)^, **u**^(*t*)^ is the controls for the system to influence next states **x**^(^*^t+^*^1)^, and $L\left(\left\{{\text{x}}^{\left(t\right)}\right\},\left\{{\text{y}}^{\left(t\right)}\right\},\left\{{\text{u}}^{\left(t\right)}\right\};\theta \right)$ is a loss function serving a specific purpose for the system to be minimized over temporal information **x**^(*t*)^, **y**^(*t*)^, and **u**^(*t*)^ along with some constraints $C\left(\left\{{\text{x}}^{\left(t\right)}\right\},\left\{{\text{y}}^{\left(t\right)}\right\},\left\{{\text{u}}^{\left(t\right)}\right\};\phi \right)$. Any of the vectors **x**^(*t*)^, **y**^(*t*)^, and **u**^(*t*)^ can be real-valued vectors or vectors of random variables such that the temporal sequences can be deterministic or a random processes adaptation. Although dimensions of *n, m, p* may be infinite in Equation (18), almost all real-life implementations are finite dimensions. Equation (18) may apply to many legacy ART approaches in different manners. In the following, we provide a brief overview for alternative ART approaches.

#### 4.1.1 Linear Feedback ARTs

Traditionally, linear feedback (loop) control systems are considered as viable implementations of ART, where most of the adaptive feedback is based on imaging information such as CT and/or MRI. Generally, there are two types of control systems: *open-loop* and *closed-loop*.

**Figure 10:**
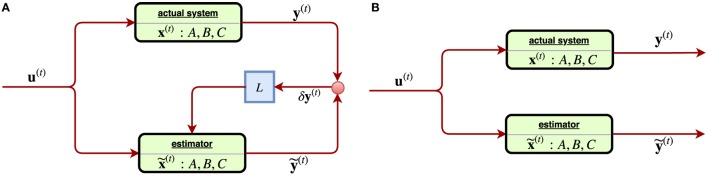
**(A)** [Left] a linear closed-loop control system, where the feedback signal of **estimation error** of the observed state *δ***y**^(*t*)^ is received through a gain (matrix) *L*. **(B)** [Right] a linear open-loop control system where the feedback signal *δ***y**^(*t*)^ was not considered. Such system tends to suffer from estimation error instability, ${\text{lim}}_{\text{t}\to \infty }$ ||δ**x**^(*t*)^|| → ∞.

With notations in Equation (18), a *linear loop control* is generally described by two sets of linear equations:

(19) $${\dot{\text{x}}}^{\left(t\right)}=A{\text{x}}^{\left(t\right)}+B{\text{u}}^{\left(t\right)},\;{\text{y}}^{\left(t\right)}=C{\text{x}}^{\left(t\right)}$$

(20) $${\dot{\tilde{\text{x}}}}^{\left(t\right)}=A{\tilde{\text{x}}}^{\left(t\right)}+B{\text{u}}^{\left(t\right)}+L\delta {\text{y}}^{\left(t\right)},\;{\tilde{\text{y}}}^{\left(t\right)}=C{\tilde{\text{x}}}^{\left(t\right)},$$

where in Equation (19), *A, B, L, C* are linear operators, **y**^(*t*)^ is the *observation* of the actual state **x**^(*t*)^, and {*u*^(*t*)^|*t* ∈ T} represents controls of the system as adaptations for the treatment of a radiotherapy. Equation (20) as a similar copy of Equation (19) describes the **estimation** ${\tilde{\text{x}}}^{\left(t\right)}$, ${\tilde{\text{y}}}^{\left(t\right)}$ of the corresponding variables **x**^(*t*)^, **y**^(*t*)^ of the system, with $\delta {\text{y}}^{\left(t\right)}\;\overset{\mathrm{def}}{=}\;{\text{y}}^{\left(t\right)}-{\tilde{\text{y}}}^{\left(t\right)}$ as the **estimation error** of the observed state and in turn shall be used as the **feedback** in the subsequent iterations. With *L* ≠ 0, the system constantly receiving the estimation error shall adjust itself accordingly, and thus such is called a **closed-loop** control system, Figure 10A.

Incidentally, in the perfect case, the three characters **x**^(*t*)^, ${\tilde{\text{x}}}^{\left(t\right)}$, ${\tilde{\text{y}}}^{\left(t\right)}$ shall coincide into one with *C* = *I*, δ**y**^(*t*)^ = 0 and thus the Equations (19) and (20) reduce to one. However, in most of cases, they tend to split. In a system, where the matrix *L* vanishes, it becomes an **open-loop** control system since any feedback signal δ**y**^(*t*)^ from the system is not considered, Figure 10B. An obvious drawback of the open-loop system is the estimation instability, which can be easily seen from Equations (19) and (20) as the quantity $\delta {\text{x}}^{\left(t\right)}\overset{\mathrm{def}}{=}\;{\text{x}}^{\left(t\right)}-{\tilde{\text{x}}}^{\left(t\right)}$ describing the **estimation error** is subject to the state equation *d/dt*(*δ***x**^(*t*)^) = *A* ⋅ δ**x**^(*t*)^ with *L* ≡ 0. The solution *δ***x**^(*t*)^ = *e^AT^*⋅δ**x**^(0)^ indicates that the error has exponential growth as time elapses such that soon an open-loop system easily becomes unreliable. On the other hand, by receiving a feedback signal due to a close-loop system (*L* ≠ 0) can improve reliability, as the evolution *δ***x**^(*t*)^ = *e*^(*A* − *LC*)*t*^⋅*δ***x**^(*t*)^ will converge by suitable choice of a **gain** *L* such that the eigenvalues |λ*_i_* (*A* − *LC*)| < 1. In a linear control problem, the control is modeled by **u**^(*t*)^ = −*K***x**^(*t*)^ with a constant matrix *K* such that Equation (20) reads:

(21) x˜˙(t)=(A−LC)x˜(t)−BK u(t)

**Figure 11:**
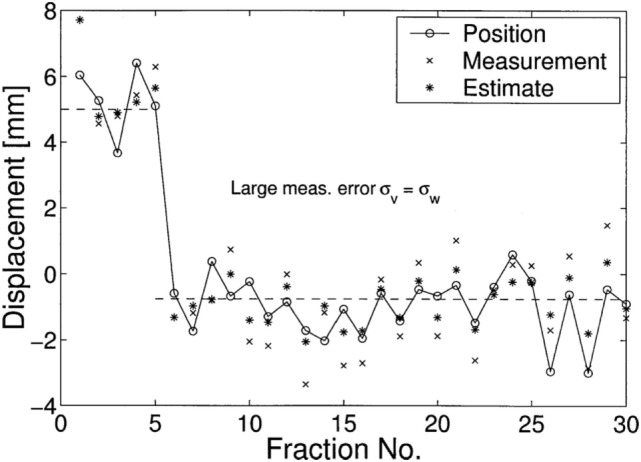
A simulated displacements (circles) of 30 fractions was demonstrated in (67), where in the first 5 fractions, *u* = 5 is used. Their results showed that on average Kalman filters (asterisks) estimations **x**˜^(*t*)^ are closer to the (unknown) displacements than the measurements **y**^(t)^ (crosses).

In control theory, one may also consider a loop-control system with small uncertainty. Typically, by considering stochasticity, the system can become more stable and robust. The (time) discretized linear control with a random process starts with extension of Equation (19) as:

(22) $$\begin{array}{ll} {\text{x}}^{\left(t\right)} & =A\cdot {\text{x}}^{\left(t-1\right)}+B\cdot {\text{u}}^{\left(t\right)}+{\text{w}}^{\left(t\right)}\\ {\text{y}}^{\left(t\right)} & =C\cdot {\text{x}}^{\left(t-1\right)}+{\text{v}}^{\left(t\right)}, \end{array}$$

where the two random processes {**w**^(*t*)^} and {**v**^(*t*)^} denote the noise of state **x**^(*t*)^ and observation **y**^(*t*)^ assumed multivariate Gaussian $N\left(\text{0},{\text{Q}}^{\left(t\right)}\right)$ and $N\left(\text{0},{\text{R}}^{\left(t\right)}\right)$, respectively. Kalman filters are then a common analysis for deriving optimal estimation of δ**x**^(*t*)^. In (67), Keller et al. established a linear stochastic closed-loop system that utilized Kalman filters (68) to derive optimal control law. They assumed an image-guided radiotherapy, which attempts to provide optimal correction strategies for setup errors, which can also take the measurement uncertainties into account. Let ${\text{x}}^{\left(t\right)}\;=\;{\text{x}}_{1}^{\left(t\right)}+{\text{x}}_{2}^{\left(t\right)}\in R^{3}$ denote the difference between the actual and planned positions of the center-of-mass of the clinical tumor volume (CTV), i.e., the daily displacement **x**^(*t*)^ containing (1) the setup error ${\text{x}}_{1}^{\left(t\right)}$ (displacement of bony structures) and (2) the organ motion (displacement ${\text{x}}_{2}^{\left(t\right)}$ with respect to the bony structures). Decompose **x**^(*t*)^ into two parts **x**^(^*^t+1^*^)^ = **u**^(*t*)^ + **w**^(*t*)^ with ${\text{u}}^{\left(t\right)}={\text{u}}_{1}^{\left(t\right)}+{\text{u}}_{2}^{\left(t\right)}$ called the **systematic component** and ${\text{w}}^{\left(t\right)}={\text{w}}_{1}^{\left(t\right)}+{\text{w}}_{2}^{\left(t\right)}$ called the **random component**, where the subindex “1” and “2” refer to setup errors and organ motion, respectively. Together, they modeled the ART displacement with a stochastic linear system:

(23) $$\begin{array}{l} {\text{x}}^{\left(t+1\right)}={\text{u}}^{\left(t\right)}+{\text{w}}^{\left(t\right)}\\ \;{\text{y}}^{\left(t\right)}={\text{x}}^{\left(t\right)}+{\text{v}}^{\left(t\right)} \end{array}$$

where **y**^(*t*)^ is the observation of **x**^(*t*)^. By defining the estimation of state **x**^(*t*)^ as ${\tilde{\text{x}}}^{\left(t\right)}\overset{\mathrm{def}}{=}\;P\left(\text{x}\;|\;{\text{y}}^{0},\ldots ,{\text{y}}^{\left(t-1\right)}\right)$ based on previous observations **y**^0^, … , **y**^(*t − 1*)^ as in Equation (20), Kalman filters are able to provide an optimal estimation of ${\tilde{\text{x}}}^{\left(t\right)}$ such that the estimation error ${\tilde{\text{x}}}^{\left(t+1\right)}\overset{\mathrm{def}}{=}{\text{x}}^{\left(t+1\right)}-{\tilde{\text{x}}}^{\left(t\right)}$ is minimal. Immediately, they derived the *optimal control law* ${\text{u}}_{c*}^{\left(t\right)}=-{\tilde{\text{x}}}^{\left(t\right)}$, which seems to be an intuitive result. A comparison was made with respect to an obvious control law that is “suboptimal” ${\text{u}}_{c}^{\left(t\right)}=-{\tilde{\text{y}}}^{\left(t\right)}$, which is merely the correction of observation itself. Subsequently, they attempted to measure the effectiveness of decisions given by Kalman filters **u**_*c*∗_ and the observation **u**_*c*_ by computing

(24) $$e\overset{\mathrm{def}}{=}\frac{\sigma_{\text{x}-\tilde{\text{x}}}^{\text{2}}}{\sigma_{\text{x}-\text{y}}^{2}}$$

where $\sigma_{\text{x}-\tilde{\text{x}}}^{2}$ and $\sigma_{\text{x}-\text{y}}^{2}$ are two residue variances of different estimation toward the state **x**^(*t*)^. One simulated result was made to demonstrate the performance of Kalman filters in predictions of stochastic linear control system, as shown in Figure 11 where a treatment of 30 fractions were simulated with the first 5 fractions, a random systematic error *u* = +5 mm and measurement noise *σ_v_* = *σ_w_* = 1 mm were imposed, which means the correction started only at the sixth fraction. Their results showed that on average Kalman filter estimations ${\tilde{\text{x}}}^{\left(t\right)}$ are closer to the (unknown) displacements than the measurements **y**^(*t*)^, where in the first fraction the estimate equals the value of the measurement.

### 4.2 Nonlinear Feedback ARTs

**Figure 12:**
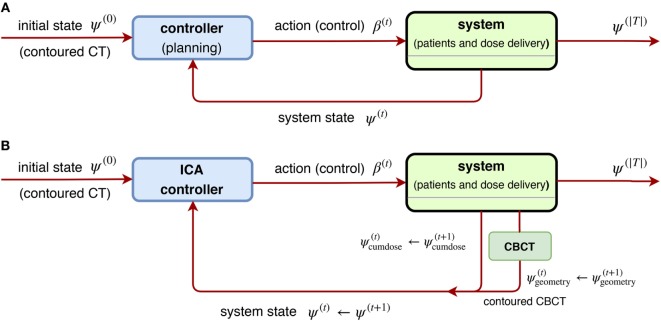
**(A)** [Left] a general scheme of a non-linear closed-loop feedback control proposed by Zerda et al. (69), where a system feedback **ψ**^(*t*)^ was received after fraction *t* ∈ *T* is completed. **(B)** [Right] block diagram of ICA algorithm proposed in (69), where the whole dose delivery history and anatomy model from daily CBCT images are considered. This is a special case of **(A)** by taking the system state $\psi^{\left(t\right)}=\left(\psi_{\text{cumdose}}^{\left(t\right)},\psi_{\text{geometry}}^{\left(t\right)}\right)$.

It is natural to consider nonlinear feedback control for ARTs due to inherent complexity. In (69), Zerda et al. developed a nonlinear closed-loop ART for treatment planning. In particular, they proposed two algorithms: *Immediately Correcting Algorithm* and *Prudent Correcting Algorithm*. With the following notation corresponding to Equation (18),

(25) $$\begin{array}{ccc} {\text{x}}^{\left(t\right)}={\text{y}}^{\left(t\right)} & \to & \psi^{\left(t\right)}=\left(\psi_{\text{ geometry}}^{\left(t\right)},\psi_{\text{cumdose}}^{\left(t\right)}\right),\\ u^{\left(t\right)}=\xi^{\left(t\right)}\left(\left\{{\text{x}}^{\left(t\right)}\right\}\right) & \to & \beta^{\left(t\right)}=\xi^{\left(t\right)}\left(\psi^{\left(t\right)}\right),\\ L\left(\left\{{\text{x}}^{\left(t\right)}\right\},\left\{{\text{y}}^{\left(t\right)}\right\},\left\{{\text{u}}^{\left(t\right)}\right\};\theta \right) & \to & \underset{v\in V}{\sum }\alpha \left(v\right){\left(D_{\text{prescribed}}\left(v\right)-\psi_{\text{cumdose}}^{\left|T\right|}\left(v\right)\right)}^{2}, \end{array}$$

where *v* ∈ *V* is a voxel under consideration, v↦α(v) is the importance factor, and the control is promoted as a nonlinear function of states, **u**^(*t*)^ = *ξ*^(^ *^t^*^)^({**x**^(*t*)^}) rather than the linear form **u**^(*t*)^ = –*K *⋅ **x**^(^*^t − ^*^1)^. With *ψ*^(*t*)^ denoting the state of the ART system, it was assumed to consists of two parts: (1) **cumulative dose** $\psi_{\text{cumdose}}^{\left(t\right)}$ after *t* ∈*T* and (2) **patient’s geometric model** obtained from conebeam CT (CBCT images) $\left\{\psi_{\text{geometry}}^{\left(t\right)}|t\in T\right\}$, where it was further assumed the geometry information interacts with the cumulative dose by the relation

(26) $$\psi_{\text{cumdose}}^{\left(t\right)}=\psi_{\text{cumdose}}^{\left(t-1\right)}+D\left(v;\left\{\beta^{\left(t\right)}|t\in T\right\},\left\{\varepsilon^{\left(t\right)}|t\in T\right\},\left\{\psi_{\text{geometry}}^{\left(t\right)}|t\in T\right\}\right)$$

with the *dose delivery* function $D\left(v;\left\{\beta^{\left(t\right)}\right\},\left\{\epsilon^{\left(t\right)}\right\},\left\{\psi_{\text{geometry}}^{\left(t\right)}\right\}\right)$ related to delivery errors {ϵ^(*t*)^}, where it is always assumed vanishing throughout the paper (69). In other words, from Equations (25) and (26), the objective of the Immediately Correcting Algorithm is to minimize the following loss:

(27) $$L\left(\beta^{1},\ldots ,\beta^{\left|T\right|}\right)=\underset{v\in V}{\sum }\alpha \left(v\right){\left(D_{\text{prescribed}}\left(v\right)-\underset{t\in T}{\sum }D\left(v;\left\{\beta^{\left(t\right)}\right\},\left\{\epsilon^{\left(t\right)}\right\},\left\{\psi_{\text{geometry}}^{\left(t\right)}\right\}\right)\right)}^{2}$$

via an optimal sequence of dose fractionation (controls) (*β*_1_, … , *β_|T|_*) to be found, and thus it is regarded as a special realization of the general scheme Figures 12A,B.

### 4.3 Stochastic ARTs

In (70), Bortfeld et al. developed a static *robust* optimization by treating the dose delivery problem of intensity modulated RT (IMRT) as a probabilistic problem with uncertainties. Using the notations in Equation (18) and letting **x**^(*t*)^ as a **breathing phase** (state) at time *t*, **u**^(*t*)^ as a **control probability function** over all breathing states, the **observed state** **y**^(*t*)^ = **x**^(^*^t^*^+1)^:

(28) $${\text{x}}^{\left(t\right)}\to x,\;{\text{u}}^{\left(t\right)}\to p\left(x\right),\;\theta \to \left\{\Delta_{v,b,x},w_{b},\gamma ,\theta_{v}\right\},$$

we arrive at the loss function and constraints proposed by Bortfeld et al.

(29) $$\begin{array}{cc} \text{minimize} & L=\underset{v\in V}{\sum }\underset{x\in X}{\sum }\underset{b\in B}{\sum }\Delta_{v,b,x}p\left(x\right)w_{b}\\ \text{subject to} & C_{1}=\underset{v\in V}{\sum }\underset{x\in X}{\sum }\underset{b\in B}{\sum }\Delta_{v,b,x}\tilde{p}\left(x\right)w_{b}\geq \theta_{v},\;\forall v\in T,\;\tilde{p}\in P_{U}\\ & C_{2}=\underset{v\in V}{\sum }\underset{x\in X}{\sum }\underset{b\in B}{\sum }\Delta_{v,b,x}\tilde{p}\left(x\right)w_{b}\leq \gamma \theta_{v},\;\forall v\in T,\;\tilde{p}\in P_{U}. \end{array}$$

Essentially, they considered the dose (to be delivered) as an expectation value following a predefined probability distribution (PDF) over all breathing phases, $D_{v,b}=E_{x}\left[\Delta_{v,b,x}\right]=\sum_{x\in X}\Delta_{v,b,x}p\left(x\right)$, where *v* ∈ *V* denotes a voxel, *b* ∈ *B* denotes a beamlet, Δ*_v,b,x_* is a matrix computed for the snapshots of the anatomy in each phase, and *θ_v_*, γ are some constants specific to the problem in question. The main purpose is to learn an optimal probability *p*(*x*) as a stochastic control overall breathing phases *x* ∈ *X* via Equation (29). The motion p.d.f. searched in the infinite-dimensional controls was actually approximated by the discretized set,

(30) $$P=\{p\in F\left(X;R\right)≅R^{\left|X\right|}|p\left(x\right)\geq 0,\underset{x\in X}{\sum }p\left(x\right)=1\}$$

such that this problem is tractable. They further required the **realization** $\tilde{p}$ of *p* in Equation (29) during a treatment to be constrained within certain error bounds ℓ and *u*:

(31) $$P_{U}:=\{\tilde{p}\left(x\right)\in P|\underset{\ell \left(x\right)}{\underset{︸}{p\left(x\right)-\underset{-}{p}\left(x\right)}}\;\leq \tilde{p}\left(x\right)\;\leq \underset{u\left(x\right)}{\underset{︸}{p\left(x\right)+\tilde{p}\left(x\right)}},\;\forall x\in U\subseteq X\}$$

As a result, the experiments by Bortfeld et al. showed that even when they allowed an unaccepted underdosage in the tumor anywhere between 6 and 11%, their proposal Equation (29) still offered same level of protection as the margin solution within 1% under dosage on average. Their approach proves that using stochastic controls helps stabilize the system with uncertainty over time. Later in (71), Chan and Mišić further improved the previous adaptive approach by extending the static probability distribution {*p*} into a temporal sequence of PDF (*p*^(1)^, *p*^(2)^, … , *p*^(^*^k^*^)^) by incorporating uncertainty set updated each time for ART, which corresponds to the sequential control {**u**^(*t*)^| *t * ∈ *T*} in Equation (18). The proposal in (71) essentially replaces the uncertainty p.d.f. *p * ∈ *P_U_* of Equation (29) by $p^{\left(k\right)}\in P_{U}^{\left(k\right)}$ iteratively to take care of patient’s breathing motions.

(32) $$\begin{array}{c} p^{\left(k+1\right)}\leftarrow p^{\left(k\right)},\;\text{with}\\ p^{\left(k\right)}\in P_{U}^{\left(k\right)}:=\left\{\tilde{p}\left(x\right)\in P|\ell^{\left(k\right)}\left(x\right)\leq \tilde{p}\left(x\right)\leq u^{\left(k\right)}\left(x\right),\;\forall x\in U\subseteq X\right\} \end{array}$$

Two versions of uncertainty updates are proposed,

(33) $$\ell^{\left(k+1\right)}=\left(1-\alpha \right)\ell^{\left(k\right)}+\alpha p^{\left(k\right)},\;u^{\left(k+1\right)}=\left(1-\alpha \right)u^{\left(k\right)}+\alpha p^{\left(k\right)}$$

(34) $$\ell^{\left(k+1\right)}=\frac{1}{k+1}\left(\ell^{\left(k\right)}+\overset{k}{\underset{i=1}{\sum }}p^{\left(i\right)}\right),\;u^{\left(k+1\right)}=\frac{1}{k+1}\left(u^{\left(k\right)}+\overset{k}{\underset{i=1}{\sum }}p^{\left(i\right)}\right)$$

where the first version is called the *exponential* smoothing update and the second is called the *running average* update. Together, Equations (29) (32), (33), or (34) constituted their proposal in (71) and suggested that their method does not require accurate information to exist before a treatment commences. Their evaluation further stressed its clinical value as it allows for the tumor dose to be safely escalated without leading to additional healthy tissue toxicity, which may ultimately improve the rate of patient survival. Subsequently, Mar and Chan (72) further proposed an extension to the adaptive robust ART mentioned above (70, 71) by adding drift component using the Lujan model (73) of patients’ breathing patterns.

Another related approach utilizing the formulation Equation (18) is found in (74), where Löf et al. developed statistical models for ART. Their design used *stochastic optimization* to handle two kinds of errors: (1) errors due to internal motion and change of organs (or tissues) and (2) errors due to the uncertainty in the geometrical setup of a patient. They attempted to compensate for the systematic errors by couch corrections and for the random error by modulation of the fluence profiles. This system was further modified by Rehbinder et al. using a linear–quadratic regulator (LQR) (75).

### 4.4 Reinforcement Learning (RL) for ART

RL is a set of machine learning algorithms that can interact with an “environment” (e.g., radiotherapy). Usually, there is a goal set for the RL, acting as an agent, to reach. Examples could be, winning a chess/board game or driving safely through a trip in an autonomous driving vehicle. Such a procedure is usually done by collecting the so-called *reward* designed by humans. RL serves as an independent machine learning area besides the common supervised or unsupervised learning mentioned earlier. RL is based on the environment defined by a Markov decision process (MDP).

An MPD is a 5-tuple (*S*, A, *P*, *γ*, *R*), where

- $S=\left\{\left(x_{1},\ldots ,x_{n}\right)\in R^{n}\right\}$ is the space of all possible states,
- A is a finite collection of all (discrete) actions,
- R:Ω→R is the reward function given on the product space Ω = *S *× A × *S*,
- *γ * ∈ [0, 1] is the discount factor, representing the importance (rewards) that propagates from the future back to the present,
- P:F → [0, 1] is a probability measure on Ω with F = 2^Ω^ the power set (*σ*-algebra) of Ω, whose probability mass function (pmf) (s,a,t)↦P(s,a,t) denotes the transition probability from state *s * ∈ *S* to another *t * ∈ *S* under an action *a * ∈ A. Consequently, this induces the condition probability
  (35) $$P_{\mathrm{sa}}\left(t\right)\equiv \text{Prob}\left(t|s,a\right)\equiv P\left(s,a,t\right)∕P\left(s,a\right),$$
  on space of next states *t* conditioned on previous state *s* and current action *a*.

As an example, in chess, each *s_i_* ∈ *S* will stand for a configuration of the chess board and action *a_i_* ∈ A corresponds to a move given by a player. The purpose of an agent in the RL is to find a sequence of actions {*a*_0_,*a*_1_, …} (acting on an initial state *s*_0_ ∈ *S*) such that a path in *S* collects maximum rewards (and hence winning the goal/game):

(36) $$s_{0}\;\overset{a_{0}}{\underset{\pi }{\to }}\;s_{1}\;\overset{a_{1}}{\underset{\pi }{\to }}\;s_{2}\;\overset{a_{2}}{\underset{\pi }{\to }}\;s_{3}\ldots$$

An agent is, by itself, a policy function *π*: *S →* A who determines an action *a* = π(*s*) under a state *s*, as described in Equation (36). There are mainly two ways to construct a policy function by *policy-based* and *value-based* methods in RL: the former parametrizes a policy function directly (76) via *π*^(^*^θ^*^)^ while the latter builds one implicitly via *Q*-functions, and hence is also called *Q*-learning. The policy-based method is usually applied in continuous controls where A≅R or large cardinality $\left|A\right|\to \infty$. In this study, we shall focus more on the *Q*-learning and its application in radiotherapy.

An optimal policy π*: *S * → A is derived from maximizing the *Q*-function in the *Q*-learning, such that $Q^{\pi^{*}}=\max_{\pi }Q^{\pi }$, where the *Q*-function is defined by evaluating the value at (*s*, *a*) ∈ *S *× A via rewards collected in all possible paths:

(37) $$Q^{\pi }\left(s,a\right)=E\left[\overset{\infty }{\underset{k=0}{\sum }}\gamma^{k}R\left(s_{k},\pi \left(s_{k}\right)\right)\left|\right.\pi ,s_{0}=s,a_{0}=a\right]$$

However, this definition Equation (37) is ideal for comprehension, yet, difficult for actual computation. A practical realization of computing the *Q*-function is via the following Bellman’s iteration, whose optimal value Q^π*^ is computed by an iterative (functional) sequence ${\left\{{\tilde{Q}}_{i}\right\}}_{i=1}^{\infty }$ instead,

(38) $${\tilde{Q}}_{i+1}\left(s,a\right)=E_{t∼P_{\mathrm{sa}}}\left[R\left(s,a\right)+\gamma \underset{b\in A}{\max}\;{\tilde{Q}}_{i}\left(t,b\right)\right].$$

Such an iteration Equation (38) is guaranteed to converge by the contraction mapping theorem (77) of the uniquely fixed point as ${\left\{{\tilde{Q}}_{i}\right\}}_{i=1}^{\infty }\to Q^{\pi^{*}}$ if *i → *∞ (78) such that

(39) $${\tilde{Q}}^{*}\left(s,a\right)=E_{t∼P_{\mathrm{sa}}}\left[R\left(s,a\right)+\gamma \underset{b\in A}{\max}\;{\tilde{Q}}^{*}\left(t,b\right)\right].$$

The calculation soon becomes intractable when either the cardinality |*S*| or |A| is large. A possible solution to this is utilizing deep learning methods for evaluating the *Q*-function proposed by Google DeepMind (79, 80), hence the name Deep *Q*-network (DQN). By taking advantages of neural networks, the convergence of the *Q*-function with Equation (38) becomes more efficient and accurate. DQN proposes ${\tilde{Q}}_{i}=Q_{\text{DNN}}^{\Theta_{i}}$, where Θ*_i_* denotes the parametrization (weights) of the DNN at *i*th iteration and requires the following loss function being optimized:

(40) $$L_{i}\left(\Theta_{i}\right)=E_{\left(s,a\right)∼\rho }\;\times \;\left[{\left(E_{t∼P_{\mathrm{sa}}}\left[R\left(t,a\right)+\gamma \underset{b\in A}{\max}Q_{\text{DNN}}^{\Theta_{i-1}}\left(t,b\right)\right]-Q_{\text{DNN}}^{\Theta_{i}}\left(s,a\right)\right)}^{2}\right].$$

In short, Equations (38) and (40), and ${\tilde{Q}}_{i}=Q_{\text{DNN}}^{\Theta_{i}}$ together makes the DQN.

### 4.5 Example: Adapting RT Plans Using Deep Reinforcement Learning

**Figure 13:**
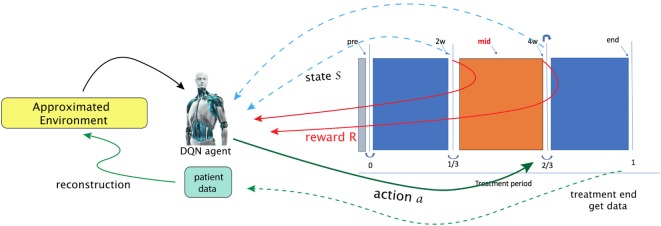
In the paper (23), Tseng et al. proposed to utilize reinforcement learning for making decisions at 2/3 period of a treatment (right solid-green arrow). A first step in their framework is to learn transition functions from the historical data of two transitions recorded (RHS figure) so that the radiotherapy environment can be reconstructed (called *approximated environment*). With the transitions simulated, a DQN agent can then search for optimal dose at each stage [figures reprinted with permission].

Using the NSCLC dataset from Section 2.5.1, we attempt to apply a DQN to provide automatic dose escalation at the 2/3 period (about 4 weeks) into a treatment as illustrated in Figure 13, where the dose escalation is the action to be submitted by the DQN. The main goal of the study is to compare the automatic decision made by the DQN to that established by a clinical protocol (81). This will be described briefly in the following, details can be consulted in (23).

That work explicitly presented a suitable MDP. In particular, a state space chosen to be useful for prediction of local control (LC) and RP2 based on the BN formalism introduced in Section 3.3.2.

By defining the state space as $S=\left\{\left(x_{1},\ldots ,x_{n}\right)\in R^{n}\right\}$ with *n* = 9 and

(41) $$\begin{array}{ccccc} x_{1}=\text{IL}4 & x_{2}=\text{IL}15, & x_{3}=\text{GLSZM}.\text{GLN}, & x_{4}=\text{GLRLM}.\text{RLN}, & \\ x_{5}=\text{MCP}1, & x_{6}=\text{TGF}\beta 1, & x_{7}=\text{Lung}\;\text{gEUD}, & x_{8}=\text{Tumor}\;\text{gEUD}, & x_{9}=\text{MTV} \end{array}$$

where *x*_1_, *x*_2_, *x*_5_, *x*_6_, *x*_9_ are cytokines, *x*_3_, *x*_4_ are of PET radiomics, and *x*_7_, *x*_8_ are doses, and here, the allowed action set will be $A=\left\{a_{1}=\text{dose}∕\text{frac}\right\}\subseteq R^{+}$. One notices that such a choice of a MDP for dose automation is not unique; there may exist other environments to attain the same or even better performance (82).

A tricky problem is that the transition probability in Equation (35) is intractable to the real world (radiotherapy environment); therefore, DNNs were utilizes to model the radiotherapy environment. Thus, a DNN provided an approximate transition probability $\tilde{P}\left(s,a;t\right):={\tilde{P}}_{\mathrm{sa}}\left(t\right):=\tilde{\text{Prob}}\left(t|s,a\right)$ modeled from the observed data, where the transition takes place $s\overset{a}{\to }t$ under action *a*. Another problem to solve in that the sample size was small relative to the DNNs. Hence, a Generated Adversarial Network (GAN) technique was used to alleviate this problem.

After proper choice of actions, A = {1, 1.1, 1.2, … , 5} Gy, and a reward function looking upon to higher LC than usual *P*^+^ baseline function:

(42) $$R\left(s\right)=\frac{1}{2}\sqrt{\text{Prob}\left(\text{LC}|s\right)}\cdot \left(1-0.8\cdot \text{Prob}\left(\text{RP}2|s\right)\right)\cdot \left(1+\mathrm{sgn}\left(17.2\%-\text{Prob}\left(\text{RP}2|s\right)\right)\right),$$

**Figure 14:**
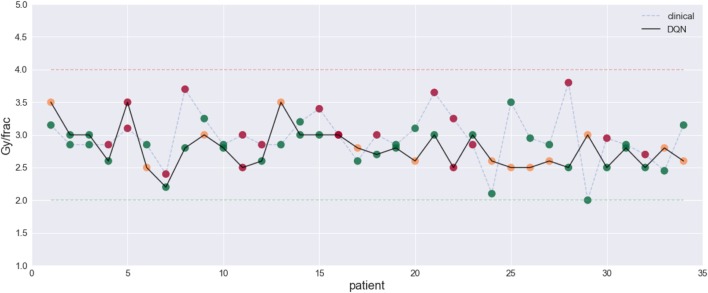
This figure visualizes the dose fraction recommended by the clinicians (blue dashed line) and the autonomous DQN (black solid line). Differences and similarities can thus be compared, with RMSE = 0.5 Gy. An evaluation of good (green dots), bad (red dots), and *potentially good* decisions (orange dots) (23) [figures reprinted with permission].

The results demonstrated the feasibility to derive automated dose levels (black solid line) that are similar to or compatible with the clinical protocol (blue dashed line) as shown in Figure 14 with the corresponding statistics shown in Table 1.

**Table 1:** Summary for the evaluation on clinicians’ and the DQN decisions extracted from (23).

Summary	Good	Bad	Potentially good
Clinicians	19 (55.9%)	15 (44.1%)	0
DQN	17 (50%)	4 (11.8%)	13 (38.2%)

## 5 Discussion

### 5.1 Statistical and Probabilistic Aspects

Here, we attempt to provide a fundamental statistical and probabilistic interpretation for sequential machine learning algorithms to help understand their roles in KBR-ART. This will be done with the specific focus on how knowledge can accumulates in such a KBR-ART system when the known information in the system is growing with time. First, we characterize the probability space as: (Ω, F, P), where F is a *σ*-algebra^1^ of a sample space Ω and $P:F\to R^{+}$ is the probability measure defined on Ω, see (83, 84). In this setting, Ω denotes the set of all possible outcomes and F as the space of all events. A (multi-dimensional) random variable **X** is a then F-measurable function $\text{X}:\Omega \to R^{n}$ on a probability space (Ω, F, *P*). Roughly speaking, the *σ*-algebra corresponds to the “information” useful (and related) to the random variable **X**. Furthermore, if {**X**^(*t*)^| *t* ∈ *T*} is a sequence of random variables (or a process), a natural *σ*-algebra induced by the process is defined by:

(43) $$U\left(t\right):=U\left(\text{X}\left(s\right)|s\in \left[0,t\right]\right)\;:=\left\{{\left({\text{X}}^{\left(s\right)}\right)}^{-1}\left(B\right)\subseteq \Omega |\forall \;\text{Borel}\;\text{set}\;B\subseteq R^{n},\;\forall s\in \left[0,t\right]\right\},$$

which is interpreted as the *history* of the process up to time *t*. Therefore, under a process {**X**^(*t*)^| *t* ∈ *T*}, one can regard the *σ*-algebra U(*t*) as accumulating information from the observed variable **X**^(s)^ along the times *s * ∈ [0, *t*]. Thus, a one liner may be best to represent the message we try to deliver:

$$\begin{array}{l} \text{a}\;\sigma \text{-algebra}=\text{information};\\ \text{a}\;\text{“growing”}\;\sigma \text{-algebra}=\text{more information coming in}. \end{array}$$

**Figure 15:**
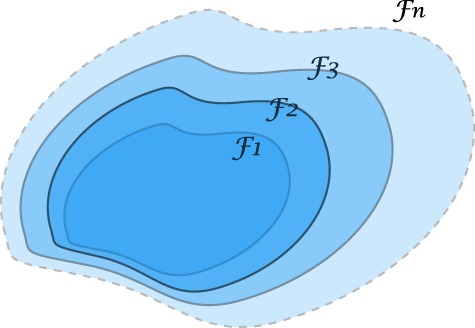
An illustration of a filtration indicating a sequence of growing *σ*-algebras, $F_{t_{\text{1}}}\subseteq F_{t_{\text{2}}}$ if *t*_1_ ≤ *t*_2_. The enlargement of *σ*-algebras reflects the accumulating information as time evolves. This provides a theoretical tool to measure the growth of knowledge in a KBR-ART design.

In fact, the idea of considering growing information, such as weather forecasting, stock pricing prediction, or daily CT changes, can be understood by a growing *σ*-algebra called a *filtration*, Figure 15. Such tool for analysis is commonly seen in quantitative finance (85, 86), which we believe it shares the same nature as a treatment in radiotherapy. The following concept describes growing (accumulating) information.

*A sequence of σ-algebras* {F*_t_*}*_t≥_*_0_ *on a measurable space* (Ω, F) *with* F*_t_* ⊆ F*is called a filtration if* $F_{t_{1}}\subseteq F_{t_{2}}$ *whenever t*_1_ ≤ *t*_2_.

The labeling index *t* is usually referred to “time” or a similar concept, where in the radiotherapy case it may be treatment fractions, stages, or phases. If we consider a filtration generated from a stochastic process via F*_t_ * = U(*t*), then, intuitively, this filtration is interpreted as containing all history available up to time *t*, but not future information available about the process. Due to this nature, a process adapted to a filtration F is also called *non-anticipating*, indicating that one cannot see into the future.

Therefore, a KBR-ART system would rely on machine learning algorithms (such as CNN, RNN, DRL, … etc.) to explore non-anticipating filtrations and to learn from accumulating knowledge or information, such as the examples givens in Sections 3.3 and 4.

To demonstrate the concept of filtrations more concretely in our setting, the following example is provided. Suppose a sequence of independent random variables {*X*^(^*^i^*^)^}*_i _*_= 1,2,3…_ denotes the *growth* in GTV size at stage *i* with E(*X*^(i)^) = *d_i_* for all *i*. If we have measured total growth up to stage *k*, i.e., *S*^(^*^k^*^)^ : = *X*^(1)^ + … + *X*^(*k*)^, we like to know what is our best guess for the growth after *n* more stages *S*^(*k*+*n*)^, given the information of the past *S*^(1)^, … , *S*^(^*^n^*^)^?

Some computation reveals that

(44) $$E\left(S^{\left(k+n\right)}|S^{\left(1\right)},\ldots ,S^{\left(k\right)}\right)=E\left(X^{\left(1\right)}+\cdots +X^{\left(k+n\right)}|S^{\left(1\right)},\ldots ,S^{\left(k\right)}\right)=S^{\left(k\right)}+\overset{n}{\underset{i=k+1}{\sum }}d_{i},$$

which indicates that the best surmise for the future value *S*^(^*^k^*^+^*^n^*^)^, given the knowledge (history) up to stage *k*, is *S*^(^*^k^*^)^ plus empirical understanding (averages), reflecting the information cease to grow after time step *k*. The computation Equation (44) relies on the following fact:

1. *If X is* F*-measurable, then* E(X|F)=X*almost surely*.
2. *If X is independent of* F, *then* E(X|F)=E(x)*almost surely*

After the above discussion of how information can be accumulated using *σ*-algebras, next, we discuss how to analyze sequential random variables from a more theoretical perspectives using time series.

#### 5.1.1 Time Series

Due to the nature of sequential data, an KBR-ART is naturally related to time series, which are applied comprehensively in forecasting, such as econometrics, quantitative finance, seismology, and signal processing, etc. Quoting from (87):

*A time series model for the observed data* {***x***^(*t*)^|*t* ∈ *T*} *is a specification of the joint distributions (or possibly only the means and covariances) of a sequence of random variables* {***X***^(*t*)^|*t* ∈ *T*} *of which* {***x***^(*t*)^} *is postulated to be a realization*.

Incidentally, a time series is a special case of **stochastic processes** {**X**^(*t*)^|*t * ∈ *T*}, where the time labeling set *T* can be an infinite set. In a very general case, a process $\left\{X^{\left(t\right)}|t\in Z\right\}$ can have *Volterra expansion*

(45) $$X^{\left(t\right)}=c+\overset{\infty }{\underset{j=0}{\sum }}\vartheta_{j}Z^{\left(t-j\right)}+\overset{\infty }{\underset{j,k}{\sum }}\vartheta_{\mathrm{jk}}Z^{\left(t-j\right)}Z^{\left(t-k\right)}+\overset{\infty }{\underset{j,k,\ell }{\sum }}\vartheta_{\mathrm{jk}}Z^{\left(t-j\right)}Z^{\left(t-k\right)}Z^{\left(t-\ell \right)}+\cdots ,$$

where high order terms can be considered. Usually, the modeling of time series is divided by two main categories, linear and non-linear methods.

In particular, there are three classes of linear models that carry practical importance, namely autoregressive models AR(*p*), the moving average models MA(*q*), and the integrated (I) models.

*(The ARMA (p,q) process with mean* μ*) The process* $\left\{X^{\left(t\right)}|t\in Z\right\}$*is called an ARMA (p,q) process if it is stationary and satisfies for all t*,

(46) $$\left(X^{\left(t\right)}-\mu \right)-\varphi_{1}\left(X^{\left(t-1\right)}-\mu \right)-\cdots -\varphi_{p}\left(X^{\left(t-p\right)}-\mu \right)=Z^{\left(t\right)}-\vartheta_{1}Z^{\left(t-1\right)}-\cdots -\vartheta_{q}Z^{\left(t-q\right)},$$

*where* $\mu ,\varphi_{i},\vartheta_{i}\in R$*and* $\left\{Z^{\left(t\right)}\right\}≃\text{WN}\left(0,\sigma^{2}\right)$*are white noise (error terms)*.

Here, the ARMA(*p*, *q*) process refers to the model with *p autoregressive* terms and *q moving-average* terms. Especially, *p* = 0 and *q* = 0 in the ARMA(*p*, *q*) process corresponds to two useful linear cases called AR(*p*) and MA(*q*) models, respectively. The aim of studying the behavior of a time series {*X*^(*t*)^} can be done *via* the analysis of the depending coefficients *φ_I_*, ϑ*_i_* and its autocorrelation function (88), which we will not go through. An interesting fact is that one can study the causality of an ARMA(*p*, *q*) process via the following fact:

*Let* {*X*^(*t*)^}* be an ARMA(p*, *q) process with φ*(*z*) : = (1 + *φ*_1_*z* + ⋯  + *φ*_1_*z^p^*)*, ϑ*(*z*) : = (1 + *ϑ*_1_*z* + ⋯  + *ϑ_q_z^q^*) *have no common zeros. Then* {*X*^(*t*)^}*is causal if and only if* $\varphi {|}_{D}\neq 0$*with* D={z∈C|∥z∥≤1}.

Thus AR(1) process with μ* * = 0 is only a simple case given by *X*^(*t*)^ = *Z*^(*t*)^ + *X*^(^*^t − 1^*^)^ from Equation (46). Since *φ*(*z*) = 1 − *φ*_1_ *z*, it follows that {*X*^(*t*)^} is causal if |*φ*_1_| < 1 and non-stationary when |*ϕ*_1_| = 1. This AR(1) case demonstrates that we may actually learn the behavior of a time series by analyzing the dependent coefficients. In fact, the heuristic AR(1) process is directly related to the Markov process due to a fact (see Proposition 7.6 in (89)). Simply stated, for a process $\left\{X^{\left(t\right)}|t\in Z\right\}$ taking values in a Borel space *S*, *Z*_1_,*Z*_2_,… are independent taking values in *E* and if there exist functions *f_t_*: *S *× *E * → *S*, t∈Z, such that *X*^(*t*)^ is recursively defined by

(47) $$X^{\left(t\right)}=f_{t}\left(X^{\left(t-1\right)},Z^{\left(t\right)}\right),\;X^{\left(0\right)}=x_{0}\in S,$$

then the process $\left\{X^{\left(t\right)}|t\in Z\right\}$ is Markov. This result Equation (47) then justifies the claim that the AR(1) is a Markov process as the transition functions simply indicate $f_{t}\left(X^{\left(t-1\right)},Z^{\left(t\right)}\right)=Z^{\left(t\right)}+\varphi_{1}X^{\left(t-1\right)}$ from Equation (47). Moreover, it is time-homogeneous since {*Z*^(*t*)^} are i.i.d. and *f_t_* is fixed across all *t*. As one recalls that the Markov process is defined under the property

(48) $$P\left({\left(X^{\left(t\right)}\right)}^{-1}\left(B\right)|U\left(s\right)\right)=P\left({\left(X^{\left(t\right)}\right)}^{-1}\left(B\right)|X^{\left(s\right)}\right)\;\left(\forall \;\text{Borel}\;B\subseteq R^{n},\;t\geq s\geq 0\right)$$

where U(*s*) is as defined in Equation (43). At the prediction level, AR(1) or Markov process then indicates that one can estimate the probabilities of future values *X*^(*t*)^ just as well as if one was aware of the entire history of the process U(*s*) prior to time *s*. The Markov property Equation (48) serves as a simplifying assumption to reduce complexities in variables involved. Therefore, it is one of our reasons to introduce the Bayesian Networks modeling based on Markov process in Section 3.2.4.

### 5.2 Comparison of Varying Data-Driven Models

There are a large number of statistical models in the area of machine learning. They can be basically divided into 3 categories: *supervised, unsupervised*, and *reinforcement learning*, where supervised models are mainly used for data *prediction*, unsupervised models are usually used to explore intrinsic data structure such as probability and location distribution, and the reinforcement learning, which we will introduce in Section 4.4 is to learn best controls within certain circumstances. All the methods introduced in Section 3.2 belong to the supervised learning category, which is the cornerstone for KBR-ART system implementation. It is essential for a KBR-ART to have a accurate model for future prediction in patients’ status, e.g., organ geometry and shape changing, whether the model is analytical or statistical. Statistical modeling is typically a handy choice over analytical one to overcome the modeling complexity involved in mechanistic realizations of radiotherapy interactions.

Comparison of the merits of several classical methods such as linear regression Section 3.2.1, Bayesian networks Section 3.2.4, decision trees, and SVMs can be found in (90–92). Generally speaking, the pros of classical data-driven models such as linear regression and Bayesian networks is that they are interpretable, numerically stable, computational efficient, and work even on small sample-sized dataset, but the cons are that they lack versatility in tasking (e.g., no one uses regressions for image segmentation or contouring) and do not possess the ability to handle complex and high variety of data, such as images, video, sequences, languages, and mixture data. For complex data such as the RT data, one can rely on more modern techniques such as deep learning, particularly DNN, CNN, and RNN-based structures. For intensive review regarding deep learning and their merits, one may refer to (42, 93). The trade-off between handling complex data and data interpretability may drive one to choose between classical and deep machine learning methods. Moreover, deep learning techniques typically require larger amount of observations compared to classical statistical learning techniques. This is a main reason that deep learning is no yet as prominent in medical and biological field compared to its current dominant in computer science and engineering. The bottom line here is that there yet no universal recognition for which classifier can do the best job in biomedicine or oncology. The development of KBR-ART is foreseeable to rely more deep learning approaches for outcome modeling and variety tasks of (image, sequential) data processing and decision-making.

## 6 Conclusion

In this study, we presented a framework for comprehensive KBR-ART design and implementation based on machine learning and explored some of its main characteristics. First, in Section 2, we analyzed the characteristics and types of features in clinical data as effective choice of data for feeding knowledge into KBR-ART. Second, in Section 3, we visited a few promising and powerful techniques of modern machine learning development, such as DNNs, CNNs, RNNs as well as the classical linear regression-type models. The KBR-ART framework we proposed here rely on machine learning techniques, which are capable of accurate prediction and sequential learning, which are the cornerstones for building up a KBR-ART system. There are three pertained questions to the design and realization of KBR-ART, which we addressed in this paper and we presented illustrative examples for each case highlighted by the application RL/BN onto a NSCLC radiotherapy dataset. In Section 4, we provided a unifying formulation in Section 4.1 for designing a KBR-ART system (Equation 18). The purpose was twofold: (1) to clearly understand the essence of previous constructed ARTs of last generation, (2) to provide a guiding principle for designing next generation algorithms.

The application of the presented technologies here provides great promise for the field of KBR-ART, yet there are still numerous challenges ahead. First, there is highly complex nature of radiation interaction with human biology that we are still trying to develop a better understanding. Second, medical datasets typically suffer from small sizes and often incomplete. Several efforts between nations and domestic institutes are being carried out to consolidate larger datasets for oncology studies, for the purpose of statistical model training and validations, but many are still in the infancy. Nevertheless, this paper still serves as a blueprint laying the foundation for the establishment and applicability of KBR-ART using modern machine learning techniques.

## Author Contributions

H-HT writes up and collects main materials related to the study; YL also collects materials related to the study; RTH plots and organizes the study; IEN directs and organizes the study.

## Conflict of Interest Statement

The authors declare that the research was conducted in the absence of any commercial or financial relationships that could be construed as a potential conflict of interest.

## Footnotes

^1^F as a collection of subsets of a set Ω is called a σ-algebra if the following three is satisfied: (1) Ω ∈ F, (2) if A∈U implies (Ω ∖ A) ∈ F, and (3) arbitrary union $A=\cup_{k=1}^{\infty }A_{k}\in F$ if *A_k_* ∈ F.
